# The First Extracellular Linker Is Important for Several Aspects of the Gating Mechanism of Human TRPA1 Channel

**DOI:** 10.3389/fnmol.2017.00016

**Published:** 2017-01-31

**Authors:** Lenka Marsakova, Ivan Barvik, Vlastimil Zima, Lucie Zimova, Viktorie Vlachova

**Affiliations:** ^1^Department of Cellular Neurophysiology, Institute of Physiology Czech Academy of SciencesPrague, Czechia; ^2^Division of Biomolecular Physics, Faculty of Mathematics and Physics, Institute of Physics, Charles UniversityPrague, Czechia

**Keywords:** TRP channel, S1–S2 linker, allyl isothiocyanate, sensor module, transient receptor potential, ankyrin receptor subtype 1

## Abstract

Transient receptor potential ankyrin 1 (TRPA1) is an excitatory ion channel involved in pain, inflammation and itching. This channel gates in response to many irritant and proalgesic agents, and can be modulated by calcium and depolarizing voltage. While the closed-state structure of TRPA1 has been recently resolved, also having its open state is essential for understanding how this channel works. Here we use molecular dynamics simulations combined with electrophysiological measurements and systematic mutagenesis to predict and explore the conformational changes coupled to the expansion of the presumptive channel's lower gate. We show that, upon opening, the upper part of the sensor module approaches the pore domain of an adjacent subunit and the conformational dynamics of the first extracellular flexible loop may govern the voltage-dependence of multimodal gating, thereby serving to stabilize the open state of the channel. These results are generally important in understanding the structure and function of TRPA1 and offer new insights into the gating mechanism of TRPA1 and related channels.

## Introduction

**Figure 1:**
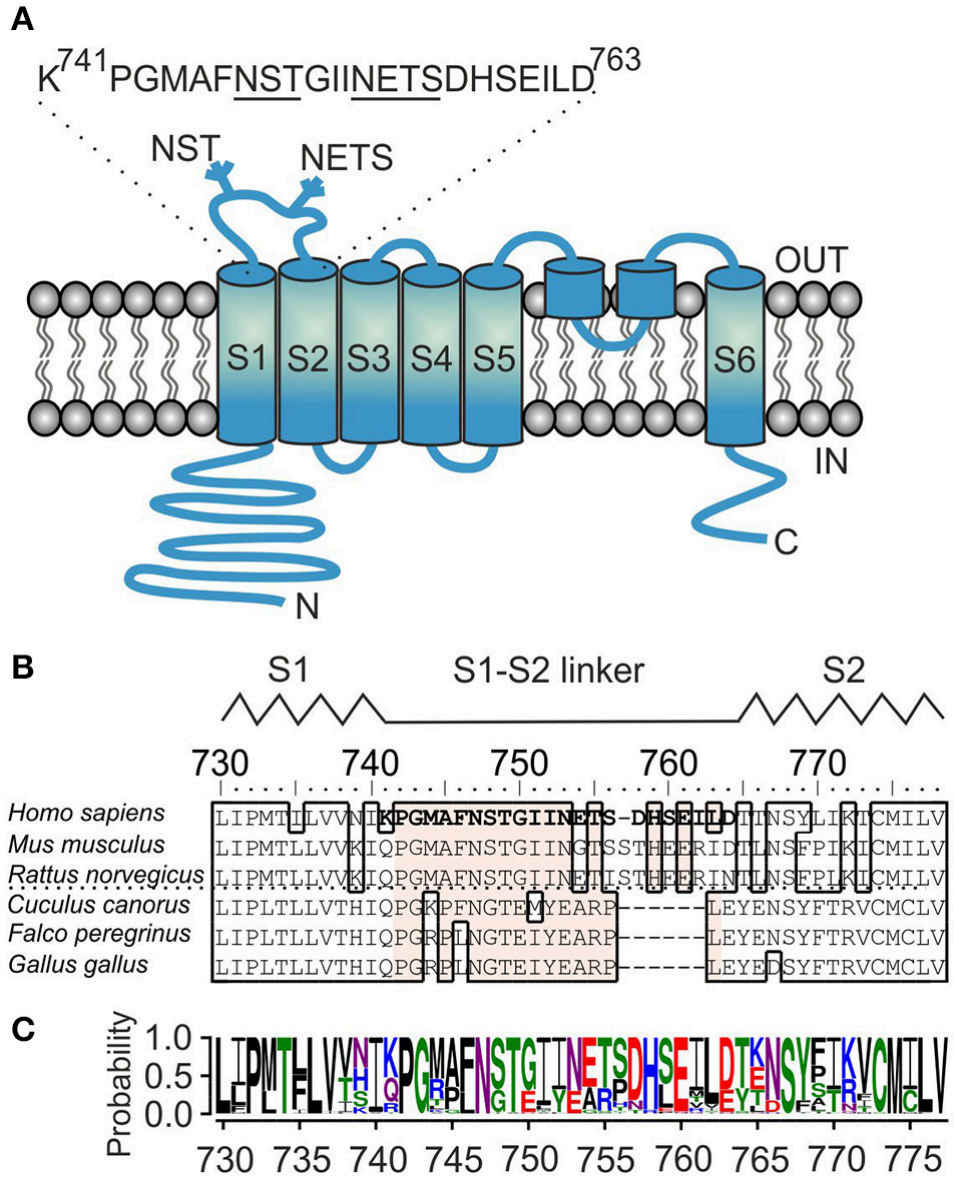
**Sequence analysis of the S1–S2 linker region of TRPA1 channel. (A)** Schematic illustration of human TRPA1 subunit with depicted position and sequence of S1–S2 linker, beginning with P742 and G743, and containing two glycosylation consensus sites ^747^NST and ^753^NETS (underlined). The extracellularly and intracellularly oriented part of the protein forms, respectively, the “upper part” and the “lower part” of the channel. **(B)** Multiple sequence alignment of S1–S2 linkers from representative TRPA1 protein orthologs. The residues mutated in this study are indicated in bold type. Conserved residues inside the S1–S2 linker across TRPA1 orthologs are depicted in beige. The interrupted line indicates sequential differences between human, rodent and bird TRPA1. Interspecies diversity from 752 to 771. The schematic location of the S1–S2 linker between the S1 and S2 helices of human TRPA1 is indicated above the alignment. **(C)** Amino-acid sequence conservation within S1–S2 linker of 57 TRPA1 proteins represented as sequence logo generated using WebLogo server (Crooks et al., 2004). The height of the particular amino acid at each position indicates its probability of occurance at that position. P742, G743, and N747 are conserved unanimously.

The transient receptor potential ankyrin 1 (TRPA1) channel responds to many environmental irritants, electrophilic compounds and proalgesic agents by opening an intrinsic ion channel gate. Specifically, in nociceptor neurons the gating of this cation channel leads to a membrane depolarization and increased intracellular calcium, which in turn modulates its own activity (Story et al., 2003; Nilius et al., 2012; Zygmunt and Hogestatt, 2014). How all the activation stimuli are conveyed through the TRPA1 protein complex to the channel's gate is still unresolved. New high-resolution structures of TRPA1 captured in a closed-pore conformation, compared with the structures of two related channels, TRPV1 and TRPV2, in open, closed and desensitized states, provide exciting clues about the common principles of TRP channel functioning (Cao et al., 2013; Liao et al., 2013; Brewster and Gaudet, 2015; Paulsen et al., 2015; Gao et al., 2016; Zubcevic et al., 2016). All these channels consist of four subunits, each containing six transmembrane segments (S1–S6) flanked by N- and C-terminal cytosolic domains, a sensor module S1–S4, and an ion conduction pore formed between S5 and S6 (Figure 1A). A comparative sequence analysis of the transmembrane regions from almost three thousand different TRP proteins reveals evolutionarily conserved features that indicate a unified model of gating mechanism (Palovcak et al., 2015). This model proposes that dynamic changes in the hydrogen-bonding network around an inherent helical distortion in the middle of S6 are translated into gating by variations in the orientation of the pore-lining residues at the hydrophobic gate. However, the statistically identifed networks of coupled residues only indicate those regions where hundreds of diverse cues that activate the large and functionally versatile family of TRP channels converge. It is known that the energetics of TRP channel gating is regulated from many interdigitated regions outside the membrane domain. Specifically, TRPA1 is unique among other TRP channels in possessing a specific modality of electrophilic activation, an even more remarkable gating promiscuity, and several distinctive structural features: its cytoplasmically located N-terminus is unusually long and its outer pore domain contains two pore helices instead of the typical solitary one present in TRPV1, TRPV2, and in the TRP-related family of voltage-gated potassium (K_V_) channels (Paulsen et al., 2015). The structures of TRPV1 in distinct functional states demonstrate a different location of the pore helix in each conformation, suggesting that it is a highly dynamic domain. In contrast, the outer pore region of TRPA1 may not be flexible enough to constitute a regulated gate due to the additional pore helix. Another apparent difference related to the presumptive gating mechanism resides in the existence of a short linker connecting S1 and S2. In TRPV1, the S1–S2 linker is static and lying atop the S1–S4 bundle (Cao et al., 2013). In TRPA1, this region extends into the extracellular space, and a poorly resolved density map in this location suggests that it possesses intrinsic structural flexibility, and thus may be actively involved in channel functioning. An extensive screening of a recombinant membrane-tethered spider toxin library recently identified a 35-residue peptide protoxin-I that antagonizes human TRPA1 through the S1–S2 linker with a high-affinity, and this interaction site is transplantable into the protoxin-I insensitive NaChBac bacterial voltage-gated sodium channel (Gui et al., 2014). Interestingly, the outer pore domain of this channel contains two pore helices like TRPA1 (Zhang et al., 2012).

In K_V_ channels, the upper part of the S1 helix forms a functionally important coevolved contacting interface between the sensor module and the pore helix of an adjacent subunit (Lee et al., 2009). The contribution of the S1–S2 linker is presumably more specific and includes stabilization of the above contacting interface (Füll et al., 2013) or N-linked glycosylation at the NXS/T consensus sequence (Zhu et al., 2003; Schwetz et al., 2010). The question of the rigidity vs. flexibility of the S1–S2 linker has been addressed in structural studies, which demonstrated that the middle section of S1–S2 contains a short helix interacting with S4 in the K_V_1.2–K_V_2.1 paddle chimera channel (Long et al., 2007), whereas this region is an extended loop in the refined structure of K_V_1.2 (Chen et al., 2010).

The amino acid sequence of the S1–S2 linker of TRPA1 is conserved in mammals but different in birds and other non-mammalian organisms (Figures 1B,C), suggesting a role for lineage-specific variations in mediating its presumably regulatory functions. We now know that from this specific region, the channel can be stabilized in its closed conformation by the specific peptide and this mode of inhibition is state-dependent (Gui et al., 2014). From the density map, we also know that this region may be intrinsically flexible. Thus, what could the functional role of the S1–S2 linker be in human TRPA1? What role, if any, does the first extracellular flexible loop play during electrophile-, voltage- and calcium-evoked channel gating? The signals generated by these stimuli likely converge into one allosteric step leading to the opening of the inner gate formed by the two hydrophobic S6 residues I957 and V961 (Paulsen et al., 2015). But how does a channel that opens in this way communicate with the upper part of the protein complex? Does it need an anchor point between the S1–S4 sensor and the pore module similar to that in structurally related voltage-gated potassium channels? Is an N-linked glycosylation at the two putative sites predicted within the S1–S2 linker involved in the gating of TRPA1? In this study, we addressed the issue of whether the opening of the TRPA1 channel may be accompanied by conformational changes near the extracellular surface. We show by modeling, electrophysiology and mutagenesis the importance of the S1–S2 extracellular linker for TRPA1 channel functioning.

## Results

### Modeling the open state of TRPA1 channel

**Figure 2:**
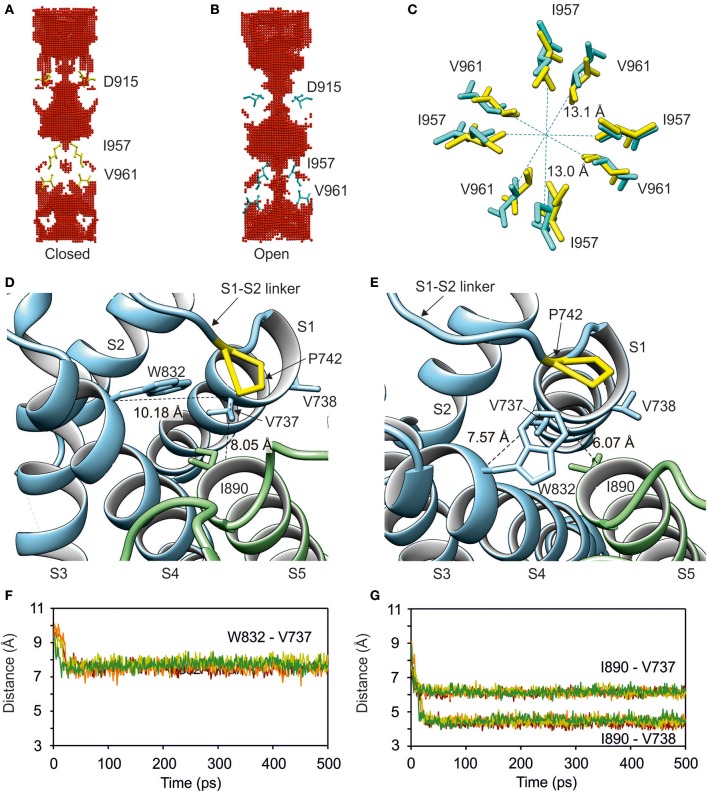
**Modeling of the open state of TRPA1 channel**. Simulated opening of the lower gate is accompanied by conformational changes in the sensor domain. **(A)** Side view of the channel cavity for closed and open state **(B)** of human TRPA1 with depicted lower gate- (I957 and V961) and upper gate-forming residues (D915) of each subunit. The gate-forming residues are shown. The channel cavity was determined using HOLLOW. **(C)** Top view of detailed changes in distances between I957 and V961 during the opening of TRPA1. In **(A–C)**, residues in yellow correspond to the closed state and cyan residues correspond to the open state. **(D)** Interface between sensor and pore domains in closed and open **(E)** state of the channel. Proline P742 (yellow, S1–S2 linker) and four hydrophobic residues V737, V738 (S1), W832 (S4) and I890 (S5) forming a “hydrophobic switch” are indicated. **(F,G)** Time course of distances between W832 (S4) and I890 (S5) from V737 and adjacent V738 (S1). See also Video 1.

To gain initial insights into the nature of the mechanical coupling between the lower gate (i.e., amino acids I957 and V961) and the sensor S1–S4 domain (i.e., amino acids S725-L850), we modeled the opening of the TRPA1 channel by using the available structures of TRPA1 in its closed conformation (3J9P; Paulsen et al., 2015) and TRPV1 in its open conformation (3J5Q; Cao et al., 2013). Over the course of Molecular Dynamics Flexible Fitting (MDFF) simulation (for a detailed description of MDFF procedure see Materials and Methods), a marked expansion of the TRPA1 pore occurred in the lower gate region (Video 1, Figures 2A,B). The distances between the alpha-carbon atoms of I957 isoleucines from diagonally positioned subunits of the channel homotetramer increased from 11.6 to 13.1 Å, and the distances between alpha-carbon atoms of V961 valines increased from 10.6 to 13.0 Å (Figure 2C).

**Figure 3:**
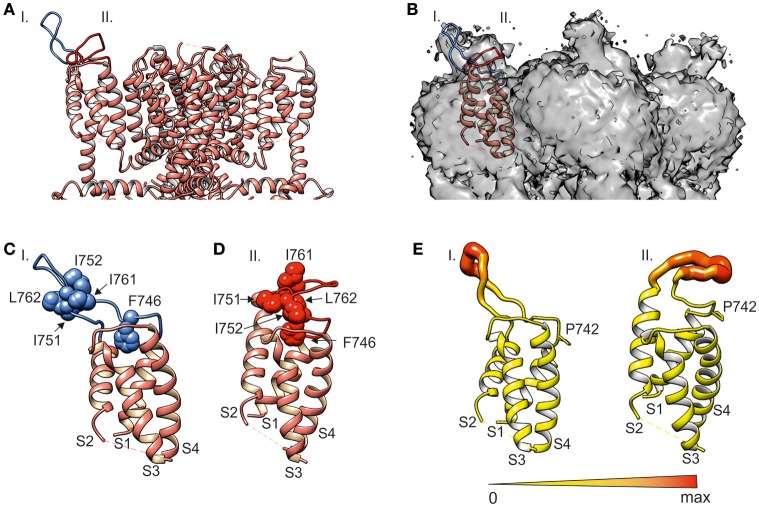
**MD simulations of S1–S4 sensor module (i.e., amino acids S725-L850) reveal two distinct conformations stabilized by hydrophobic interactions. (A)** Ribbon diagram of the transmembrane domain (i.e., amino acids N722 to A971) of human TRPA1 channel with two predicted S1–S2 linker conformations (I and II, blue and red) added to TRPA1 structure 3J9P (Paulsen et al., 2015, depicted in salmon color). **(B)** Electron density map EMD-6267 of TRPA1 superimposed on the S1–S4 sensor domain and two predicted S1–S2 linker conformations I and II (blue and red). **(C,D)** Details of the S1–S4 sensor domain shown in **(A)**. **(C)** Conformation I (blue) represents the stable hairpin stabilized by hydrophobic residues. **(D)** Conformation II (red) interacts with the upper part of the sensor module via hydrophobic residues. Phenylalanine F746 is situated in the middle of the upper part of the S1–S4 sensor domain. **(E)** The extent of S1–S2 linker flexibility for both conformations represented as a putty representation of the protein colored according to average B-factor was prepared in Chimera using the Render by Attribute menu under Tools and Structure Analysis menu. The diameter and color of the S1–S2 linker are scaled to the maximum B-factor. Orange to red colors and a wider tube indicate a region with a higher average B-factor, whereas shades of yellow and a narrower tube represent a region with a lower average B-factor. Proline P742 at the beginning of the S1–S2 linker is shown.

The expansion of the lower gate was accompanied by a conformational change and switching of amino acids on the extracellular side, where the S1–S4 sensor approaches the S5–S6 pore domain of an adjacent monomer. Helices S1, S4, and S5 come into close contact—specifically, the amino acids of helices S1 (V737), S4 (W832), and S5 (I890) (Figures 2D–G). Proline at position 742 situated at the beginning of the S1–S2 linker is in close contact with this hydrophobic switch and mediates contacts between the S1–S4 sensor module and the pore-forming domain. Among the hydrophobic amino acids within the S1–S2 linker, phenylalanine F746 (shown in Figure 3) was constantly oriented into the protein bulk and seemed to significantly contribute to stabilization of the sensor module. The results from this MDFF simulation indicate that the extracellular S1–S2 linker may be physically coupled with the conformational changes in the lower gate, and may thus be an important regulator of TRPA1 channel gating.

### Structural modeling of S1–S2 linker indicates two distinct conformations stabilized by hydrophobic interactions

To gain further mechanistic insight into the putative role of the S1–S2 linker (amino acids S748-D763), we performed a 500-ns molecular dynamic (MD) simulation for the S1–S4 sensor module (i.e., amino acids S725-L850) in aqueous solution (see Materials and Methods for details). The resulting models were structurally aligned with the published TRPA1 structure 3J9P and compared with the density map of TRPA1 EMD-6267 (Paulsen et al., 2015). The simulation revealed two general conformations of the S1–S2 loop (Figure 3A). A remarkably stable hairpin conformer (conformation I) was found over time from 262 to 412 ns, fitting part of the density well (Figure 3B). In the rest of the MD simulation (from 37.5 to 112.5 ns), the S1–S2 loop was conformationally less pronounced (conformation II) and corresponded to the other part of the electron density map for this region.

The MD simulation indicates that the electron density maps of TRPA1 show a mixture of two conformers. The first one, corresponding to the hairpin structure, appears to be significantly stabilized by hydrophobic interactions between amino acids I751, I752, I761, and L762 (Figure 3C). The second one is a mixture of sub-conformers, wherein the above-mentioned hydrophobic amino acids interact with the upper part of the sensor module S1–S4, including F746 (Figure 3D). Our structural simulations thus substantiate the intrinsic flexibility of the S1–S2 linker (Figure 3E) and also indicate that this region is capable of adopting conformations that can effectively influence the changes carried out by S1–S4 helices during channel gating, presumably through steric interactions of the hydrophobic part of the linker.

### Isoleucines I751 and I752 regulate the voltage-dependent activation of TRPA1

**Figure 4:**
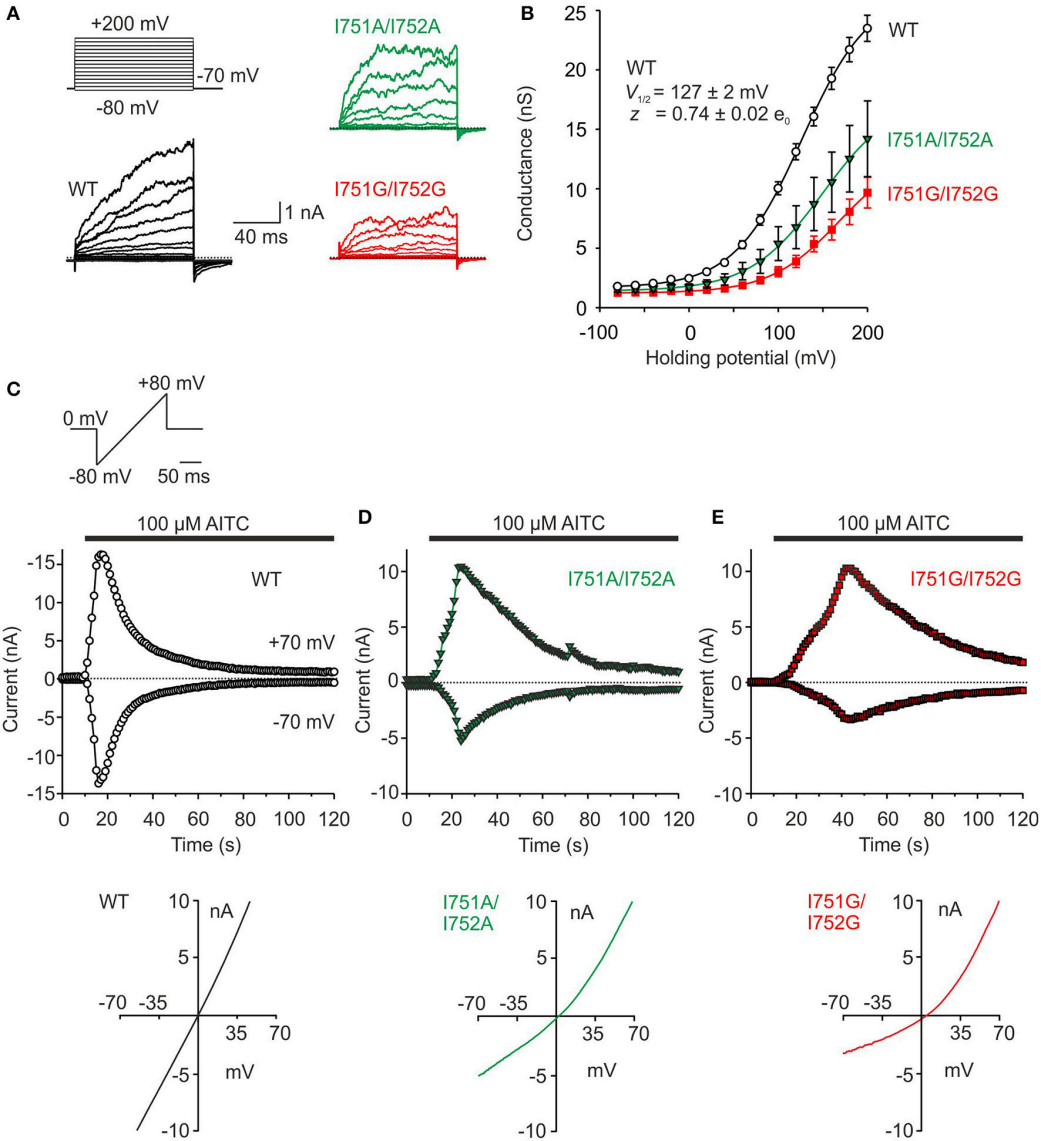
**Mutations of isoleucines 751 and 752 alter voltage and AITC sensitivity of TRPA1. (A)** Representative currents from wild type (WT, black), I751A/I752A (green) and I751G/I752G (red) mutant TRPA1 in response to indicated voltage step protocol (holding potential −70 mV; 100 ms voltage steps from −80 mV to +200 mV, increment 20 mV) recorded in control extracellular solution ~1 min after whole-cell formation. **(B)** Average conductances of wild type (WT, white circles, *n* = 121), I751A/I752A (green triangles, *n* = 22) and I751G/I752G (red squares, *n* = 31) mutants obtained from the voltage step protocols as in **(A)**. Depolarizing voltage activated human TRPA1 to a maximal amplitude of 4.8 ± 0.2 nA (measured at +200 mV). Voltage-dependent gating parameters were estimated by fitting the conductance G = I/(V-V_rev_) as a function of test potential V to the Boltzmann equation: G = [(G_max_-G_min_)/(1+exp (−zF(V-V_1/2_)/RT))] + G_min_, where z is the apparent number of gating charges, V_1/2_ is the half-activation voltage, G_min_ and G_max_ are the minimum and maximum whole cell conductance, V_rev_ is the reversal potential, and F, R, and T have their usual thermodynamic meanings. Compared to the wild type, the double mutant channels exhibited lower voltage sensitivity, a drop in maximal conductances and a shift in V_1/2_ values toward higher depolarizing potentials (V_1/2_ = 154.6 ± 4.9 mV, *z* = 0.98 ± 0.11 e_0_, *n* = 13 for I751A/I752A and V_1/2_ = 152.1 ± 4.9 mV, *z* = 0.87 ± 0.05 e_0_, *n* = 20 for I751G/I752G). Data represent mean ± SEM. **(C)** Time course of representative current responses of wild type, I751A/I752A **(D)** and I751G/I752G **(E)** mutant channels evoked by 100 μM AITC in control extracellular solution during a continuous ramp protocol (from −80 to +80 mV, every 1 s). The current responses were assessed at −70 and +70 mV. The horizontal bar above the records indicates the period of AITC application. In cells expressing wild-type TRPA1 channels, AITC produced robust (7.7 ± 0.9 nA; *n* = 18; at +70 mV) and rapid current responses at both positive and negative membrane potentials (time of peak 8.4 ± 1.0 s at +70 mV) followed by immediate desensitization. In contrast, both the I751A/I752A and I751G/I752G mutants exhibited much slower kinetics of AITC-induced responses (time of peak 23.2 ± 3.8 s, *n* = 5 and 29.8 ± 3.0 s, *n* = 4; at +70 mV). Notably, both the double mutants exhibited a significant reduction in AITC-induced amplitudes selectively at negative membrane potentials, as is clearly visible in the bottom current-voltage (I-V) relationships obtained from the cells shown above at the peak of AITC responses.

The hydrophobic cluster that substantially contributes to the two general conformations of the S1–S2 linker includes a prominent couple of adjacent isoleucines I751 and I752. To explore their presumptive structural significance, we substituted both the residues simultaneously with a smaller and less hydrophobic alanine (I751A/I752A) or an even smaller and flexible glycine (I751G/I752G). The voltage sensitivity was examined by using a voltage step protocol from −80 mV up to +200 mV in extracellular control solution (Figure 4A). For wild-type TRPA1, the Boltzmann fit gave a half-maximal activation voltage (*V*_1/2_) of 127.2 ± 1.9 mV and an apparent number of gating charges (*z*) of 0.74 ± 0.02 e_0_ (*n* = 121) which agrees with the previously published data (Sura et al., 2012). Compared to wild-type TRPA1, both the double-mutant channels exhibited lower sensitivity to voltage, and their half-activation potential *V*_1/2_ was shifted toward more positive membrane potentials (Figure 4B). To test their chemical sensitivity, we used a protocol in which whole-cell membrane currents were measured in the presence of the full electrophilic agonist allyl isothiocyanate (AITC, 100 μM) in extracellular control solution. The membrane potential was ramped up each second from −80 to +80 mV (Figure 4C). The double mutants exhibited much slower kinetics of AITC-induced responses and a significant reduction in currents at negative membrane potentials (Figures 4D,E and Supplementary Figure 1A). As was previously shown, current-voltage (*I*-*V*) relationships of wild-type TRPA1 responses to AITC are linear at the peak, and become less linear as they inactivate (Wang et al., 2008; Kremeyer et al., 2010). In the I751A/I752A mutant, and even more remarkably in I751G/I752G, the *I*-*V* relationships exhibited strong outward rectification at the peak of AITC responses (Figures 4D,E, graphs below current traces). The glycine at positions 751 and 752 caused more pronounced effects than alanine, which indicates that the volume of residues and local conformational flexibility may be important for full activation of the channel–specifically at hyperpolarized voltages. Consistent with this finding, the double mutants exhibited lower basal activity and much smaller average responses than wild-type TRPA1 when we measured the AITC-induced activity by a Ca^2+^-imaging technique (F_340_/F_380_; Supplementary Figures 1B,C).

### I751G/I752G mutant exhibits different rectification pattern

**Figure 5:**
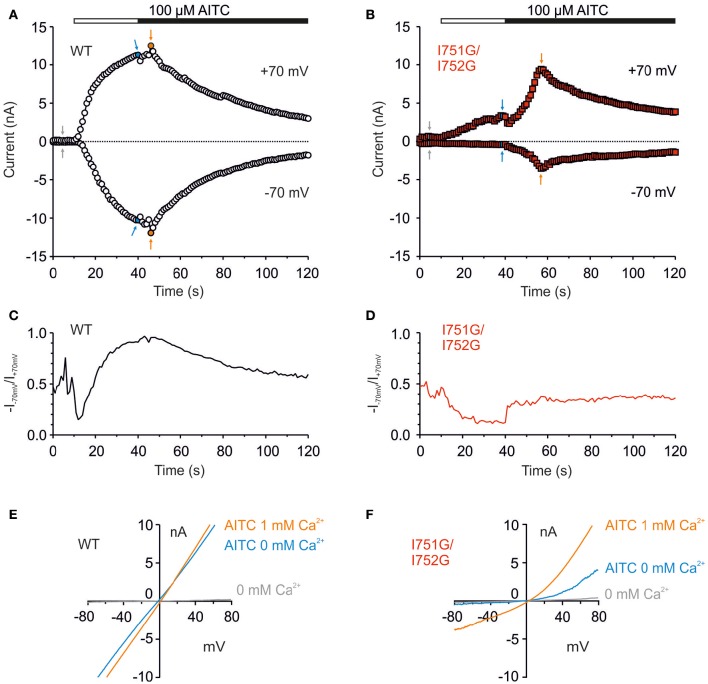
**The I751G/I752G double mutation affected AITC currents in a voltage-dependent manner. (A)** Representative whole-cell recordings from HEK293T cells expressing wild type and I751G/I752G mutant **(B)** during activation with 100 μM AITC without Ca^2+^ (white horizontal bar) and in the presence of 1 mM Ca^2+^ in extracellular solution (black horizontal bar). A continuous ramp protocol was used (from −80 to +80 mV, every 1 s). Graphs were assessed at +70 and −70 mV. The mutant exhibited slower kinetics of Ca^2+^-induced potentiation of AITC responses (time of peak 12.6 ± 2.1 s, *n* = 11, in contrast to 3.9 ± 0.5 s, *n* = 64 in wild type at +70 mV) but the rate of inactivation remained unchanged. **(C)** Changes in rectification ratio (*R* = −I_−70mV_/I_+70mV_) for the cell expressing wild-type and **(D)** double mutant TRPA1 over the same time period as in **(A,B)**. Double mutant was not able to reach full activation at the peak of AITC response, neither in the absence (max R = 0.19) nor in the presence of Ca^2+^ (max R = 0.50; *n* = 11). **(E,F)** I-V relationships constructed from currents shown in **(A,B)** at times indicated with arrows; gray (basal), blue (after a 30-s application of AITC without Ca^2+^) and orange (AITC with 1 mM Ca^2+^ in extracellular solution).

The above experiments were done in the presence of extracellular control solution which contained 1 mM external Ca^2+^. Calcium ions permeating through the TRPA1 channel exert bimodal effects, both potentiating the current and accelerating its inactivation (Wang et al., 2008). To examine the contribution of Ca^2+^, we used a protocol in which whole-cell membrane currents were measured first in the absence of extracellular Ca^2+^ and in the presence of the full agonist AITC (100 μM, 30 s). Ca^2+^ at a concentration of 1 mM was then added to the bath solution. As before, intracellular Ca^2+^ was buffered to low levels with 5 mM EGTA in the patch pipette to assess the effects of permeating Ca^2+^ ions (Wang et al., 2008). This protocol enabled us to explore not only the AITC sensitivity of the channels to an electrophilic agonist that leads to the persistent activation of TRPA1 (Hinman et al., 2006; Macpherson et al., 2007), but also to permeating Ca^2+^ that modulates the channel through various mechanisms (Doerner et al., 2007; Zurborg et al., 2007). In wild-type TRPA1, AITC in the absence of Ca^2+^ elicited large membrane currents at both membrane potentials which came close to saturation in 30 s (Figure 5A). The subsequent addition of Ca^2+^ evoked a mild potentiation followed by inactivation. In contrast, the I751G/I752G mutant exhibited small responses to AITC in the absence of Ca^2+^ at depolarizing potentials, and no currents were seen at negative potentials (Figure 5B). Calculations of the rectification index (*R* = −current at −70 mV/current at +70 mV) showed clear differences between wild-type and I751G/I752G channels when exposed to AITC (Figures 5C–F), indicating that the mutation modified the channel's behavior in a voltage-dependent manner.

### Impact of the particular physico-chemical properties of residues replacing I751 and I752

**Figure 6:**
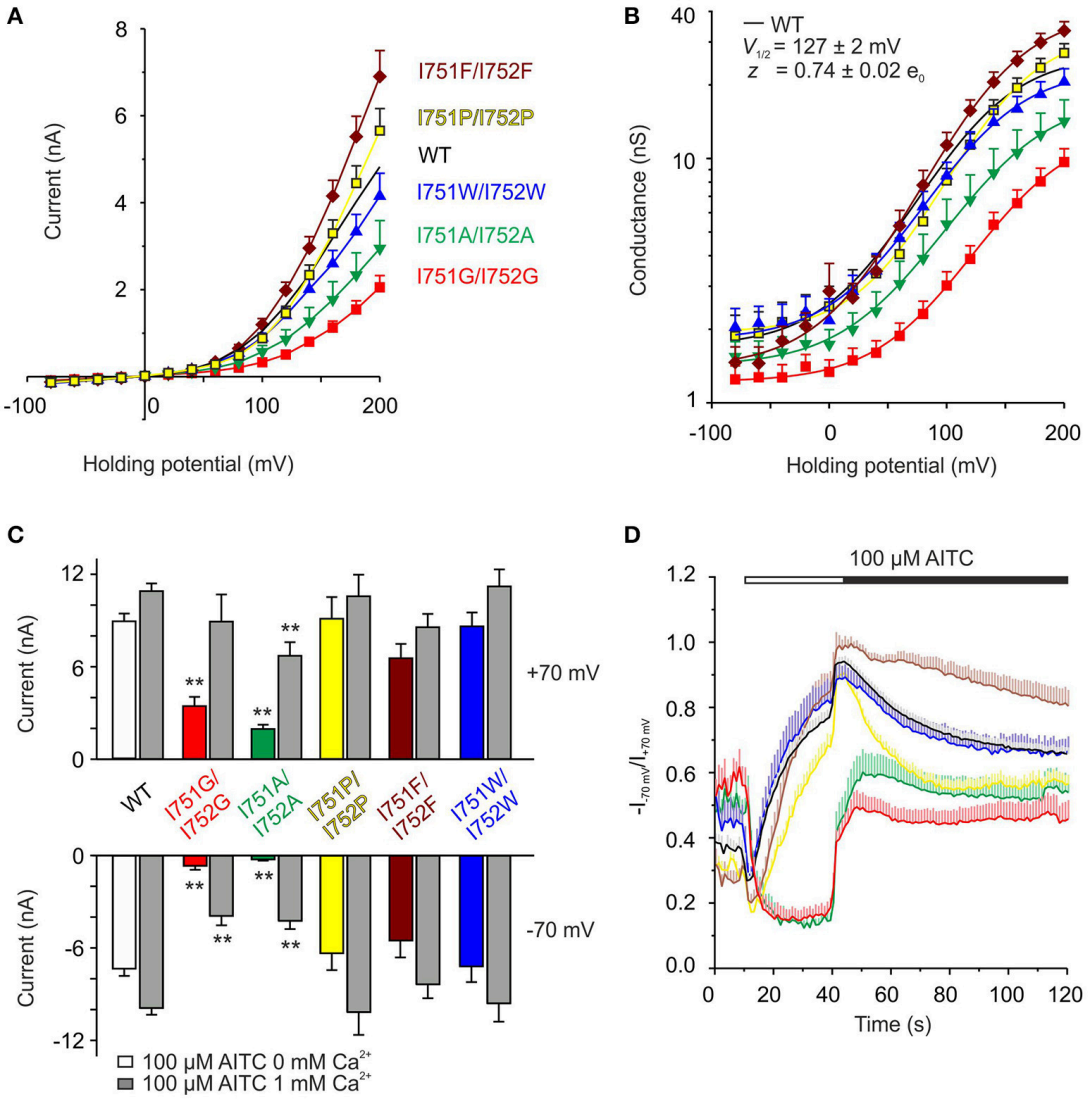
**The precise size and hydrophobicity of amino acids at position of isoleucines 751 and 752 are critical parameters for the voltage activation of TRPA1. (A)** Average current-voltage relationships of wild type (WT, *n* = 121) shown as a black line and indicated double mutants of isoleucines shown as colored curves (I751A/I752A, *n* = 22, green; I751G/I752G, *n* = 31, red; I751P/I752P, *n* = 25, yellow; I751F/I752F, *n* = 17, brown; I751W/I752W, *n* = 24, blue) obtained from steady-state of current responses induced by voltage protocol as in Figure 4A (100 ms voltage steps from −80 mV to +200 mV, holding potential −70 mV; increment 20 mV), measured in extracellular control solution. Data are presented as mean ± SEM. **(B)** Average conductances of individual double mutants and WT obtained as in A from voltage-step protocols (see Figure 4A) using equation *G* = *I*/(*V*-*V*_rev_), fitted as in Figure 4B. *V*_1/2_ = 146.1 ± 3.1 mV, *z* = 0.81 ± 0.04 e_0_ for I751P/I752P, *V*_1/2_ = 149.4 ± 3.4 mV, *z* = 0.75 ± 0.04 e_0_ for I751F/I752F, and *V*_1/2_ = 123.5 ± 5.3 mV, *z* = 0.78 ± 0.06 e_0_ for I751W/I752W. Colors as in **(A)**. **(C)** Summary graph of maximum AITC currents of wild type (WT, *n* = 63) and isoleucine double mutants (I751A/I752A, *n* = 11; I751G/I752G, *n* = 8; I751P/I752P, *n* = 12; I751F/I752F, *n* = 10 and I751W/I752W, *n* = 10) from experiment as in Figure 5A, in the absence of Ca^2+^ (colored bars) and in the presence of 1 mM Ca^2+^ (peak, gray bars), measured at +70 mV and −70 mV. The asterisks indicate a significant difference from wild-type TRPA1, ^**^p < 0.001. **(D)** Average changes in rectification ratio (*R* = −I_−70mV_/I_+70mV_) for wild-type TRPA1 (black line) and indicated double mutants, colored as in **(A)**. The applications of AITC without Ca^2+^ (white horizontal bar) and in the presence of 1 mM Ca^2+^ (black horizontal bar) are indicated above. Data represent mean ± SEM, n as in **(C)**.

To further determine the specificity of the regulation of TRPA1 by the S1–S2 linker, we examined the importance of the physico-chemical properties of various amino acid residues substituted at positions I751 and I752. We explored residues with different hydrophobicities or volumes and measured the sensitivity of the double mutants to depolarizing voltages (Figures 6A,B) and to AITC in the absence or presence of Ca^2+^ (Figures 6C,D and Supplementary Figure 2). There were differences among mutants and wild-type channels in terms of their voltage-dependent gating and the kinetics of their AITC-induced responses. I751P/I752P and I751F/I752F did not differ from the wild type in the peak of AITC responses at both potentials (Figure 6C), however, the time courses of rectification exhibited different kinetics (Figure 6D). Whereas, the former double mutant exhibited a slower onset of the rectification index in AITC and a faster and more complete inactivation at negative potentials after the addition of Ca^2+^, the latter mutations led to opposite effects: a faster onset and a slow and incomplete Ca^2+^-dependent inactivation. When comparing the functional impacts of mutations with the properties of the substituting amino acids (Supplementary Figure 2F), it becomes evident that a specific combination of the volume, hydrophobicity and flexibility imparted by the isoleucines at positions 751 and 752 is critical for the voltage-dependent gating of TRPA1.

### I751G/I752G mutant is distinctly blocked by external calcium

**Figure 7:**
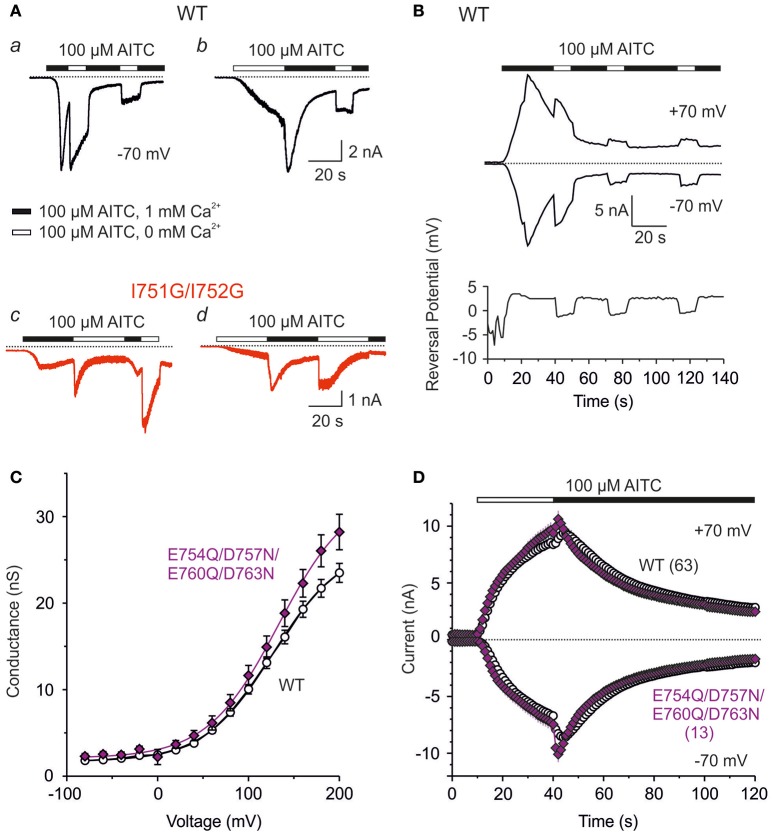
**External Ca^2+^ reversibly block human TRPA1 channel in a state-dependent manner and distinctly block I751G/I752G mutant. (A)** Representative current responses recorded from a HEK293T cell expressing wild-type TRPA1 (**A*a*** and ***b***; black traces) or I751G/I752G mutant (***c*** and ***d***; red traces), stimulated with 100 μM AITC in changing Ca^2+^ concentration at a constant potential of −70 mV. The application of AITC without (white horizontal bar) and with Ca^2+^ ions (1 mM, black horizontal bar) are indicated above the current traces. Similar effects were seen in 3–4 cells for each construct. **(B)** Time course of representative whole-cell currents of HEK293T expressing wild-type TRPA1 during voltage ramp protocol (from −80 to +80 mV, every 1 s) and application of 100 μM AITC in the control extracellular solution containing 1 mM Ca^2+^ (black horizontal bar) and after several removals of Ca^2+^ from the bath solution (white horizontal bar), measured at −70 mV and +70 mV. Below, the time course of reversal potential for the same cell and the same time period. **(C)** Neutralization of four negative charges within S1–S2 linker changed neither the voltage nor AITC response of TRPA1 channel. Average conductances of wild type (white circle, *n* = 121) and E754Q/D757N/E760Q/D763N quadruple mutant (violet diamond, *n* = 17) obtained from voltage step protocols as in Figure 4A. Lines are best fits to a Boltzmann function as described in Materials and Methods. **(D)** Time course of average whole-cell current responses induced by 100 μM AITC in the absence of Ca^2+^ (white horizontal bar) and subsequent addition of 1 mM Ca^2+^ to the bath solution (black horizontal bar), during continuous ramp protocol (from −80 to +80 mV, every 1 s), measured at −70 and +70 mV. Current responses through wild type are depicted as white circles and E754Q/D757N/E760Q/D763N mutant as violet diamonds. All data represent mean ± SEM (n indicated in brackets).

To further explore how the S1–S2 region may contribute to chemical activation apart from any allosteric effects of depolarizing voltage, we measured currents from wild-type and I751G/I752G channels in response to AITC applied either in the absence or presence of 1 mM Ca^2+^ at a constant voltage of −70 mV. Two different protocols were used. In the first case, 100 μM AITC in a Ca^2+^-containing bath solution was applied to the wild type or to the I751G/I752G double mutant. As shown in Figure 7, wild-type TRPA1 responded to AITC with robust and fast inward currents followed by rapid inactivation (Figure 7A*a*). Removing Ca^2+^ from the extracellular solution immediately (in < 1 s) unblocked monovalent cation flow through the channels (Karashima et al., 2010). If the external Ca^2+^ was removed once again 20 s later, the recovery from the block was not complete and a slowly progressing component of inactivation was apparent. In the second protocol, TRPA1 was first activated by AITC without Ca^2+^, which evoked a slowly rising response and then Ca^2+^ was added 30 s later (Figure 7A*b*). The addition of Ca^2+^ caused a rapid increase in currents followed by Ca^2+^-dependent inactivation. The subsequent removal of external Ca^2+^ only partially recovered the channels from inactivation. This finding demonstrates that Ca^2+^, in addition to its well-described potentiating and inactivating effects, reversibly blocks the flow of monovalent cations. The extent of this block is state-dependent, so the less inactivated channels are more blocked. A similar blocking effect of external Ca^2+^ was also apparent for a protocol in which the membrane potential was ramped up each second from −80 to +80 mV (1 V/s) (Figure 7B). Under the same protocols, I751G/I752G exhibited qualitatively different behavior. The channels appeared to be blocked by Ca^2+^ right from the start, and were only transiently unblocked by removing external Ca^2+^ (Figure 7A*c*). By using a protocol in which AITC was initially applied without Ca^2+^, we observed very slowly developing responses (Figure 7A*d*). The addition of 1 mM Ca^2+^ potentiated and inactivated the channels as in the wild type. However, in contrast to the wild-type, the channels recovered fully from the block after the removal of Ca^2+^ from the bath (compare Figures 7A*b*,*d*), indicating a perturbed gating equilibrium in favor of the closed state. The removal of external Ca^2+^ had immediate and reversible effects on TRPA1, which suggests an external site of Ca^2+^ action. The S1–S2 linker of human TRPA1 has four negatively charged amino acids which could be involved in Ca^2+^ binding. However, when we neutralized all of them (E754Q/D757N/E760Q/D763N), we did not see any significant changes in either the voltage or the chemical responsiveness (Figures 7C,D). These experiments thus more suggest an important contribution of the hydrophobic residues in the S1–S2 linker in Ca^2+^-dependent regulation and further support their active role in regulating the gating equilibrium of the TRPA1 channel.

### S1–S2 linker mediates TRPA1 activation at hyperpolarized potentials

**Figure 8:**
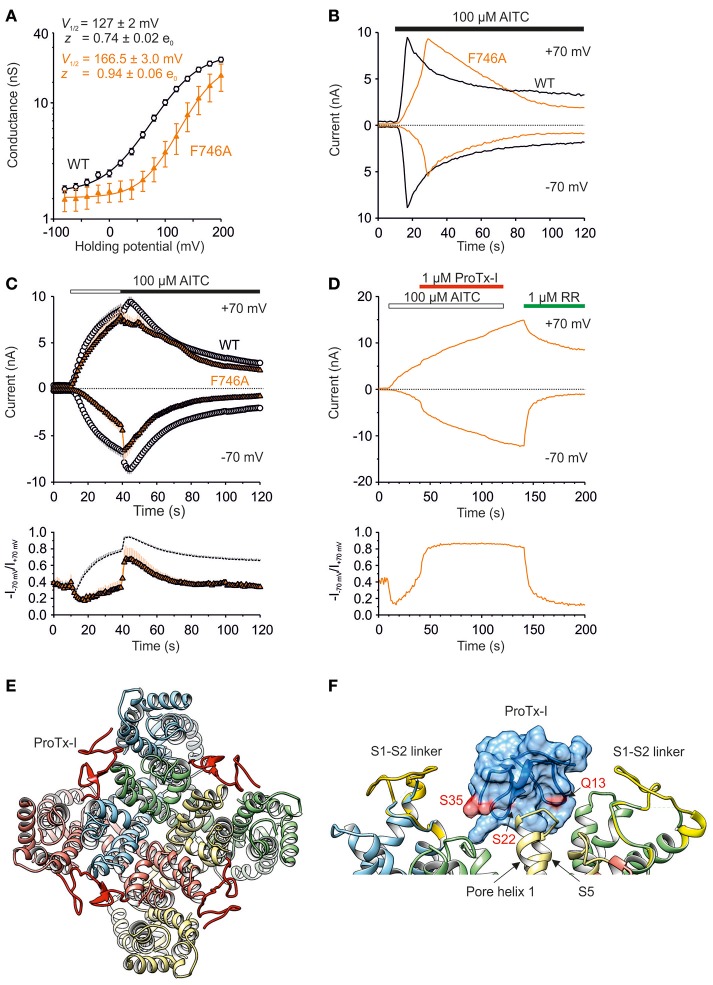
**S1–S2 linker mediates TRPA1 activation at hyperpolarized potentials. (A)** Logarithmic scale of conductance-voltage relationship for wild type (white circles, *n* = 121) and F746A mutant (orange triangles, *n* = 12) obtained from voltage step protocol as in Figure 4A (holding potential −70 mV; 100 ms voltage steps from −80 mV to +200 mV, increment 20 mV). The mutation strongly shifted the *V*_1/2_ value to more positive potentials (166.5 ± 3.0 mV, *n* = 10; *p* < 0.001) and significantly increased the gating charge to 0.94 ± 0.06 e_0_ (*p* < 0.05). Voltage-dependent gating parameters were estimated as in Figure 4B. Data represent means ± SEM. **(B)** Representative currents from HEK293T cells expressing wild type (black traces) or F746A mutant (orange traces) at −70 and +70 mV, measured as in Figure 4C. The AITC-induced currents through F746A recorded in the extracellular control solution exhibited a delayed time of peak (19.6 s ± 3.6 s, *n* = 6; at +70 mV) and markedly reduced responses at negative membrane potentials. AITC application is indicated above. **(C)** Time course of average whole-cell currents through wild type (white circles, *n* = 63) and F746A mutant (orange triangles, *n* = 7) evoked by AITC, measured at −70 and +70 mV. The application of 100 μM AITC without Ca^2+^ (white horizontal bar) and subsequent addition of 1 mM Ca^2+^ (black horizontal bar) are indicated above. Below, average rectification of currents shown above. Changes in rectification ratio plotted as a function of time, calculated as absolute value of current at −70 mV divided by current at +70 mV (*R* = −I_−70mV_/I_+70mV_). **(D)** Representative current responses of wild-type TRPA1 induced by application of AITC (100 μM, white bar), subsequent addition of protoxin-I (ProTx-I, 1 μM, red bar) and ruthenium red (RR, 1 μM, green bar), all in the absence of Ca^2+^. The application protocol is indicated above. Below, rectification of current responses shown above. Changes in rectification ratio plotted as in **(C)**. Protoxin-I application prevented rectification (R close to 1). Similar effecs were seen in two other cells. **(E)** Protoxin-I (ProTx-I) docked to the TRPA1 structure (3J9P) and refined with MD. ProTx-I is in red, Q13, S22 a S35 involved in binding to TRPA1 (Gui et al., 2014) are indicated. Conformation II of the S1–S4 sensor modules with the S1–S2 linkers (S748-D763, yellow) were structurally aligned. Nitrogens from POPC bilayer are shown in blue. **(F)** Detail from **(E)** showing binding surface (pink) mapped onto the protoxin-I structure, amino acid residues involved in binding to TRPA1 are indicated. Subunits are distinguished by gray shades.

The molecular simulations revealed that, during in-silico opening, phenylalanine F746 is constantly oriented toward the sensor module and forms stable interactions with the very beginning of the S1–S2 linker and the upper part of the S1–S4 bundle (Figure 3D). In the TRPA1-related TRPM3 channel, this region forms an alternative ion permeation pathway, distinct from the central pore, which can be uncovered by the specific mutation of an aromatic residue at the top of S4 (Vriens et al., 2014). Although the upper crevice of the S1–S4 sensor is likely to be unsolvated in TRPA1 (Palovcak et al., 2015), F746 is located right in its center and interacts, among others, with W832 from upper S4. This led us to test whether replacing F746 with a small residue would cause changes in the channel's functioning or would enable the formation of an ion permeation pathway analogous to that in TRPM3. Figure 8A shows that the mutation F746A strongly shifted *V*_1/2_ to more positive potentials and significantly increased the gating charge. Compared to the wild type, the AITC-induced currents recorded in the extracellular solution containing 1 mM Ca^2+^ exhibited a delayed time of peak and markedly reduced responses at negative membrane potentials (Figure 8B). This reduction was also observed when AITC was applied in the absence of external Ca^2+^ (Figure 8C). At positive potentials and in the presence of Ca^2+^, the maximum AITC currents were no different from those of wild-type TRPA1. All these observations suggest an active role of F746 in voltage-dependent gating, instead of physically plugging a putative alternative pore. Hydrophobic packing around F746 in the upper part of the S1–S4 module seems to maintain the activity of the channel at negative membrane potentials.

We next hypothesized that protoxin-I, which inhibits wild-type TRPA1 by binding primarily to the S1–S2 linker but also to other parts of the S1–S4 extracellular surfaces (Gui et al., 2014), might help to explain the functional impact of the F746A mutant. Surprisingly, instead of inhibiting, 1 μM protoxin-I increased the AITC-induced activity of F746A at negative membrane potentials and the rectification ratio (*R* = −I_−70mV_/I_+70mV_) became almost immediately constant and close to 1 (Figure 8D), indicating that the binding of protoxin-I may compensate for the intrinsically lower voltage sensitivity of the mutant. This result further supports our predictions that there is a functional relationship between the S1–S4 module and the S1–S2 linker that is substantially involved in the voltage-dependent gating of TRPA1. To predict potential sites of interaction between the transmembrane part of TRPA1 (i.e., amino acids A685-K989) and protoxin-I (Figures 8E,F), we used molecular docking and molecular dynamics simulations (see Materials and Methods for details) taking into account the homology of protoxin-I with the structurally similar vanillotoxin DkTx that binds the pore region of TRPV1 and traps it in its open state (Cao et al., 2013; Bae et al., 2016). The results of this MD simulation showed that, indeed, protoxin-I is capable of binding the S1–S4 sensor of TRPA1. In addition, the simulation confirmed that the S1–S2 linker is an extracellular loop and its conformational preferences predicted above were most likely not affected by the absence of phospholipids. By mapping the known active surface of protoxin-I (Gui et al., 2014) onto the upper part of the S1–S4 sensor and the predicted set of conformations of the S1–S2 linker, we hypothesize that the linker could act as a pivot component in the gating apparatus of TRPA1, which moves against the channel core to affect (or stabilize) its conformations.

**Figure 9:**
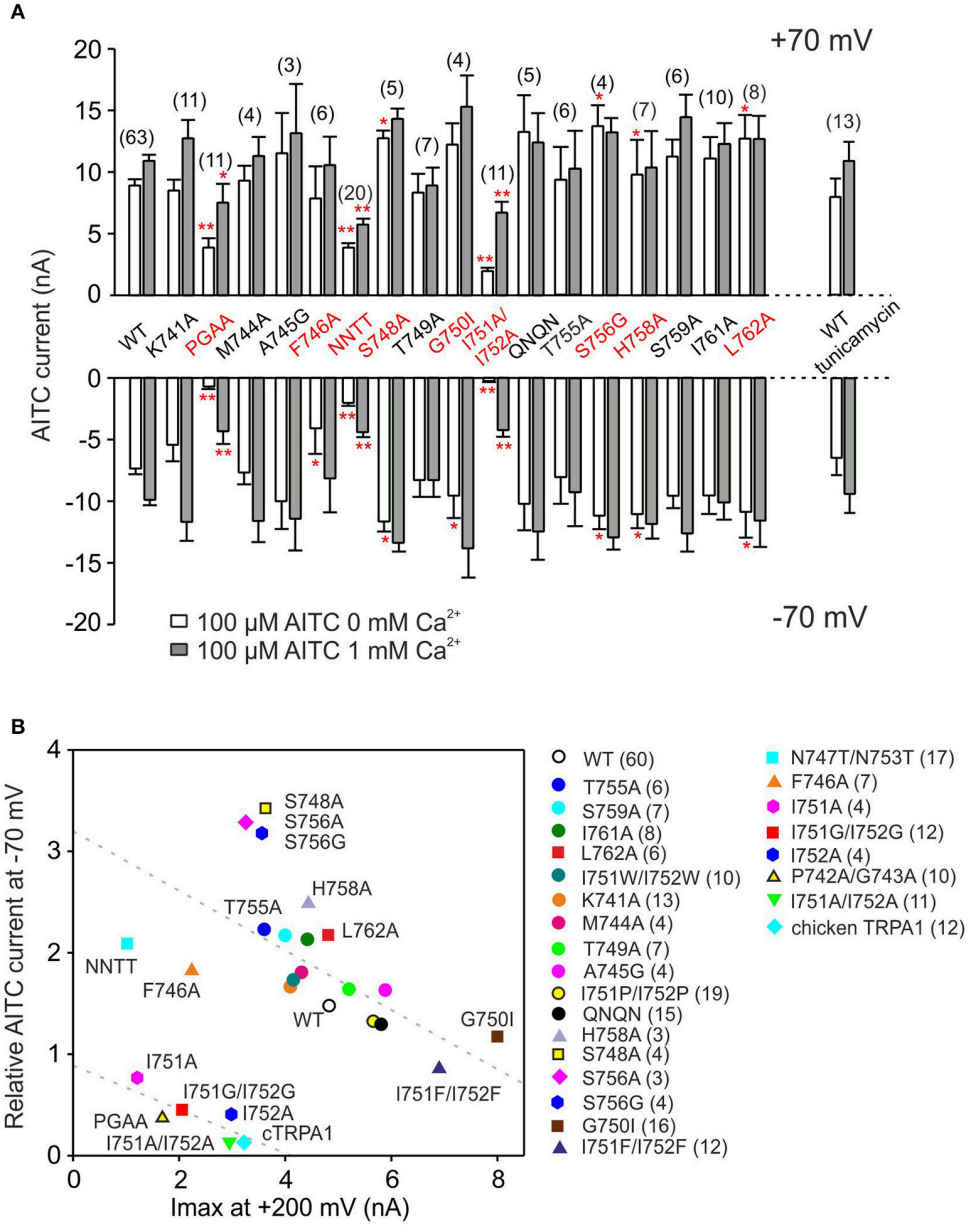
**Summary of the effects of point mutations in the S1–S2 linker on AITC-induced currents. (A)** Summary bar graph of average AITC-induced current responses from experiments as in Figure 5A, measured at +70 mV and −70 mV in the absence (white bars) and in the presence of 1 mM Ca^2+^ (gray bars) for wild-type TRPA1 (WT) and mutant channels. NNTT indicates the N747T/N753T double mutant; QNQN indicates the E754Q/D757N/E760Q/D763N quadruple mutant. n are indicated in brackets for each mutant. Asterisks indicate a significant difference from the wild type, ^*^p < 0.05, ^**^p < 0.001. Data represent mean ± SEM. **(B)** Correlation of the maximum currents induced by depolarizing voltage (axis x; at +200 mV; see voltage step protocol in Figure 4A) against inward currents induced after 30-s exposure to AITC in the absence of Ca^2+^ at −70 mV divided by their maximum currents induced at +200 mV (y axis; I_AITC_ at −70 mV/I_max_ at +200 mV). NNTT indicates the N747T/N753T double mutant; QNQN indicates the E754Q/D757N/E760Q/D763N quadruple mutant. Mutations indicated as circles exhibited phenotype not significantly different from wild type. The minimal number of measured cells is depicted in the graph. Data are means ± SEM.

To obtain the complete function-based characteristics of the S1–S2 linker, we next measured the responses to voltage and AITC of several additional mutants that were designed to affect their presumable contribution to flexibility, hydrophobicity, charge and N-glycosylation (Figure 9). The relationship between two experimental parameters for voltage-dependent gating were summarized for all the mutants in Figure 9B: the maximum amplitude of currents induced by depolarizing voltage at +200 mV and the relative inward currents induced after 30-s exposure to AITC in the absence of Ca^2+^, measured at −70 mV. This relationship enables us to qualitatively correlate the changes in voltage-dependent gating measured under control conditions with the changes in allosteric coupling between agonist interaction site(s) and the voltage-dependent opening of the channel gate. The graph revealed four different phenotypes of mutants: (1) most of the mutants were negatively correlated with similar values to wild-type TRPA1, from the less voltage-dependent T755A to the gain-of-function mutant G750I, (2) mutations at serines S748 and S756 increased the relative amplitude of the AITC-induced inward currents, (3) the single or double alanine mutants of P742, G743, I751, and I752 had very low voltage-dependent responses at +200 mV, and even lower amplitudes of AITC-induced inward currents and (4), F746A and the double mutant N747T/N753T were clearly set apart from the other constructs, indicative of their specific functional roles.

### Mutations at two predicted N-glycosylation sites decrease TRPA1 expression

There are two predicted N-glycosylation sites on the S1–S2 extracellular linker of human TRPA1 according to NetNGlyc 1.0 Server (^747^NSTG and ^753^NETS), however, there are no consistent reports about the glycosylation state of this channel (Deering-Rice et al., 2015; Egan et al., 2016). In our expression system, the N747T/N753T mutant obviously differed from other less responsive mutants in that its AITC responses were much less rectified at negative membrane potentials (Supplementary Figures 3A–D). To determine whether these changes might stem from changes in N-glycosylation, the cDNA of GFP-tagged TRPA1 or its double mutant N747T/N753T were transiently transfected into HEK293T cells and cell lysates were incubated in the absence or presence of peptide-N-glycosidase F (PNGase F) at 37°C for 3 h (Supplementary Figure 3E). Immunoblot analysis showed that the double mutant achieved in average only 77% of the total wild type protein expression (Supplementary Figure 3F), which roughly corresponds to the reduction in currents. The dominant molecular weight of the double mutant treated with PNGase F was ~140 kDa, which was no different from the wild type. Most likely, in our expression system, TRPA1 does not undergo substantial N-linked glycosylation and the effect of the double mutation N747T/N753T could be attributed to structural changes in the S1–S2 loop that are crucial for proper expression. This is further supported by our two findings: (1) Other mutations disrupting the N-glycosylation consensus sites (S748A and S756A/G) rendered a distinct phenotype of channels with significantly smaller responsiveness to depolarizing voltage but larger and significantly less rectifying AITC currents (Figure 9B). (2) When the transiently transfected HEK293T cells were grown for 24 h in the presence of 5 μg/ml tunicamycin, a drug that prevents the N-glycosylation of proteins, the amplitudes and the time course of rectification of TRPA1-mediated AITC-responses were no different from control measurements (Figure 9A, Supplementary Figures 3C,D).

### S1–S2 may distinctly modulate voltage-dependent gating in mammals and birds

**Figure 10:**
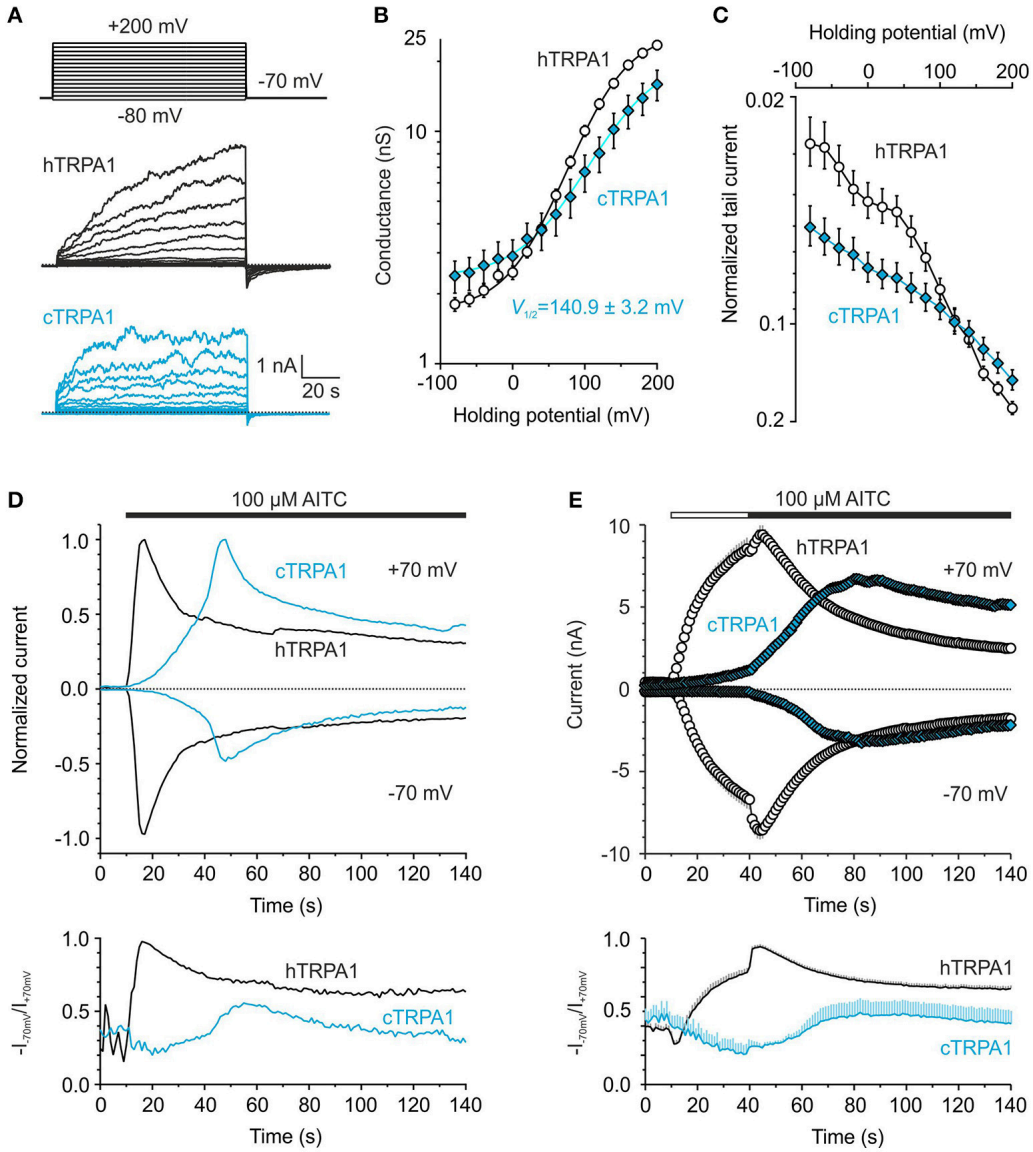
**Chicken and human TRPA1 exhibit different voltage and chemical gating. (A)** Representative whole-cell current traces from human (hTRPA1) and chicken TRPA1 (cTRPA1) in response to indicated voltage step protocol (holding potential −70 mV; 100 ms voltage steps from −80 mV to +200 mV; increment +20 mV) recorded in control extracellular solution ~1 min after whole-cell formation. **(B)** Average conductance-voltage relationship obtained from voltage step protocols as in **(A)**. A logarithmic scale was used to compare the energetic effects of hTRPA1 (*n* = 121) and cTRPA1 (*n* = 31) at hyperpolarized voltages. Compared to human TRPA1, chicken TRPA1 exhibited smaller current responses evoked by depolarizing voltages (3.2 ± 0.5 nA; *p* < 0.001; at +200 mV) and a shift in conductance-to-voltage relationships (G-V) toward more depolarizing potentials (*V*_1/2_ = 140.9 ± 3.2 mV; *p* < 0.001; could be estimated from 16 out of 31 cells). **(C)** Average tail currents measured 1.5–1.6 ms after returning to the holding potential −70 mV normalized to the maximal current at +200 mV. **(D)** Representative currents evoked in response to 100 μM AITC in control extracellular solution measured at −70 and +70 mV, normalized to their maximal amplitudes at +70 mV. The AITC currents through cTRPA1 were slower (time of peak 67.3 ± 16.7 s at +70 mV), they reached a peak amplitude comparable with that obtained with hTRPA1 at +70 mV (7.7 ± 2.7 nA, *n* = 3; *p* = 0.58) but a moderately lower peak at −70 mV (−3.2 ± 1.6 nA, compared to −6.0 ± 0.6 nA, *n* = 18 in hTRPA1; *p* = 0.118). AITC was applied for the time indicated by the horizontal bar. Below, changes in rectification index (*R* = −I_−70mV_/I_+70mV_) plotted as a function of time for the same cell. **(E)** Time courses of average currents induced by 100 μM AITC in the absence of Ca^2+^ (30 s, white horizontal bar) and subsequent addition of 1 mM Ca^2+^ (black horizontal bar), measured at −70 and +70 mV. Below, average changes in rectification index for the cells shown above. The responses of hTRPA1 are depicted as white circles and black lines (*n* = 63) and cTRPA1 as blue diamonds and blue lines (*n* = 7). The data represent mean ± SEM.

The results from molecular modeling support the previous hypothesis that the gating of TRP channels could share a conserved mechanism involving an external contacting interface between the sensor and the pore of an adjacent subunit (Palovcak et al., 2015). The proline P742 is a structurally unique residue that is part of the S1–S2 linker whose sequence from P742 to T755 is highly conserved in mammals but differs from bird species (Figure 1B). If the S1–S2 linker of human TRPA1 is involved in gating, we should expect differences in the voltage-dependent gating properties of chicken TRPA1. Compared to human TRPA1, chicken TRPA1 exhibited a shift in the conductance-to-voltage relationship toward more depolarizing potentials (Figures 10A,B). The less voltage-dependent phenotype of chicken TRPA1 was also detectable as a dramatic reduction in tail currents during repolarization to −70 mV from previous depolarizing steps and by an increase in the voltage-independent component (Figure 10C). The AITC currents through the chicken TRPA1 reached a peak amplitude comparable with that obtained with human TRPA1 at +70 mV, but were much slower and exhibited a lower peak at negative potentials (Figure 10D, Supplementary Figure 4). The maximum rectification R_max_ for chicken TRPA1 measured at the peak of AITC-induced responses was ~0.5, which means that the channels were far from being fully activated at hyperpolarized potentials (Figure 10D, lower plot). Also, a 30-s application of AITC in the absence of Ca^2+^ and then the addition of Ca^2+^ to the bath solution, did not lead to full activation at negative potentials (R_max_ = 0.49) (Figure 10E). Apart from the proximal N- and distal C-terminal regions, the lowest sequence similarity between chicken and human TRPA1 is constrained to the S1–S2 linker, which exhibits only a 35% similarity, compared to the rest of the protein (79%). Therefore, it seems plausible that amino acid differences in the extracellular S1–S2 region underlie, at least partially, the observed functional difference between human and chicken channels.

## Discussion

The current understanding of how TRP channels gate is based on the recent structural analysis of the TRPA1, TRPV1, and TRPV2 channels (Cao et al., 2013; Liao et al., 2013; Paulsen et al., 2015; Zubcevic et al., 2016). From comparisons among these structures, it appears that the overall gating mechanisms may be partially shared (Palovcak et al., 2015), however, the precise regulatory domains differ. The high sequence variability among species observed in the S1–S2 extracellular linker, surrounded by the largely conserved transmembrane core, is one of the distinctive features of TRPA1, suggesting that this domain may have a unique biological function. This study was ultimately motivated by this enigmatic feature and has six important implications for understanding the gating mechanisms of TRPA1.

First, our results demonstrate that the human TRPA1 channel has evolved an external regulatory domain that must specifically interact with the upper part of the sensor module to stabilize the channel's open state when activated at hyperpolarized potentials. The predicted conformations of the S1–S2 region include structurally important hydrophobic residues that must be properly positioned relative to the sensor as well as to the pore module. The hairpin structure (Figure 3C) likely corresponds to the closed state of the channel, which seems to be more accessible for the specific toxin (Gui et al., 2014). The other conformation (Figure 3D) helps to stabilize the open state, as inferred from our findings that hydrophobic interactions are important for the proper functioning of the channel. This view is also supported by our observation that replacing serines S748 and S756 with hydrophobic residues improved AITC-induced responses (Figure 9).

Second, the impaired voltage-dependent gating in the chicken TRPA1 orthologue, strikingly resembling impaired responses through the F746A mutant of human TRPA1 (compare Figures 8B, 10D), raises the possibility that the S1–S2 linker of chicken TRPA1 lacks the specific contacts required for electromechanical coupling between the sensor and the pore. Given the high degree of sequence variability among species, we hypothesize that this extracellular domain is critical for the species-specific regulation of TRPA1 voltage-dependent gating.

Third, our results may help to interpret the recent cryo-electron microscopic structure of TRPA1. The original density map indicated a poorly resolved α-helical structure in the S1–S2 linker that extends into the extracellular space (Paulsen et al., 2015). We show that this region is intrinsically flexible and represented by two main conformers, one of them dedicated to stabilizing the open channel by interactions with the upper part of the sensor. In this conformer, we indeed observed that structural alterations during MD simulation (from 305 to 356 ns) involved unwinding/rewinding of a helical turn in the region G750–I752.

Fourth, the results from our MDFF simulations indicate that the extracellular S1–S2 linker is physically coupled with the conformational changes in the lower gate, and may thus be an important regulator of TRPA1 channel gating. A number of lines of evidence presented here indicate that there is substantial communication between the activation gate and the upper part of the S1–S4 sensor module. The evidence that mutation at F746 can be rescued by a specific high-affinity peptide binding the S1–S2 linker provides a strong argument in favor of this view.

Fifth, our results demonstrate that the key effect of mutations in the S1–S2 linker (I751G/I752G) is in stabilizing the gate to decrease the voltage-dependent opening of the channel. The transient character of inhibitory effects of calcium that regulates the currents through the channel at hyperpolarized potentials is a further finding that supports the involvement of the S1–S2 region in the direct regulation of TRPA1 gating (Figure 7A). We demonstrated that the four negatively charged amino acids, E754, D757, E760, and D763, located at the extracellular surface of the channel are not important for the voltage-, agonist-, and Ca^2+^-dependent gating and are not involved in ion permeation through the channel. This conclusion is further supported by a recent comprehensive mutagenesis study by Christensen et al. (2016) that calculated electrostatic contributions of acidic residues in the channel vestibule and found that the only acidic residues that serve to attract permeant ions are near or within the pore.

Sixth, we note that the two presumed consensus N-glycosylation motifs on the S1–S2 extracellular linker are most likely not subjected to N-linked glycosylation, although their mutations influence its insertion into the plasma membrane. This is in contrast to the related TRPC3, TRPC6, TRPV5, TRPV6, and some K_V_ channels, whose activities are tightly regulated from the S1–S2 linker via glycosylation (Dietrich et al., 2003; Chang et al., 2005; Schwetz et al., 2010).

A recent comprehensive analysis of TRP channel transmembrane sequences identifies the conserved and coevolved networks of residues, suggesting a shared general allosteric mechanism underlying the polymodal gating (Palovcak et al., 2015). Our study extends this view and demonstrates that the most divergent regions may dominate, play a fundamental role in tuning the voltage-dependent gating and determine the rectification properties under conditions that resemble those experienced by the TRP channels in their native environment.

## Materials and methods

### Molecular dynamics simulations of the open state of the TRPA1 channel

The opening of the TRPA1 channel was modeled by using the available structures of TRPA1 in its closed conformation (3J9P; Paulsen et al., 2015) and TRPV1 in its open conformation (3J5Q; Cao et al., 2013). We applied the Molecular Dynamics Flexible Fitting (MDFF) method for combining high-resolution atomic structures with cryo-electron microscopy (cryo-EM) maps, that results in atomic models representing the conformational state captured by cryo-EM (Trabuco et al., 2008, 2009). In fact, we followed standard procedure using software packages, modules and keywords described in detail in “MDFF Tutorial” (http://www.ks.uiuc.edu/Training/Tutorials/). The original electron density map of TRPV1 (Cao et al., 2013) contains structural information about the N- and C-termini, which have a significantly different arrangement in TRPA1. Therefore, we used only the transmembrane portion of TRPV1 (i.e., amino acids K431 to S693 from 3J5Q) and generated its simulated electron map by interpolating the atomic number of each atom onto a 3-D grid and low-pass filtering it to the desired resolution (5 Å) using the software package VMD (Humphrey et al., 1996). The transmembrane part of TRPA1 (i.e., amino acids N722 to A971) was then rigid-docked into the resulting electron density map by means of the software package SITUS (Wriggers et al., 1999) to gain a starting simulated system for subsequent refinement by means of MDFF. SITUS uses for rigid docking the CoLoRes (contour low resolution docking) algorithm emulating *in silico* the simultaneous registration of surface and volume features (that is at the heart of visual docking as performed by an electron microscopist). A six-dimensional search using fast Fourier transforms is used to rapidly scan the rigid-body degrees of freedom of a probe molecule relative to a fixed target density map as described in detail in Chacón and Wriggers (2002). MDFF takes advantage of the NAMD's grid forces feature, through which an arbitrary external potential defined on a 3-D grid can be added to a molecular dynamics simulation. Here, the simulated TRPV1 electron map served to generate (again using VMD; Humphrey et al., 1996) additional potential U_EM_ that guided the transmembrane part of TRPA1 (parametrized using the CHARMM force field; Best et al., 2012) to the open conformation in the MDFF simulation produced using the NAMD software package (Phillips et al., 2005). HOLLOW was used for a “casting” of the central pore of TRPA1 by filling the voids, channels and pockets with dummy atoms defined on a grid (Ho and Gruswitz, 2008). Further details of the modeling procedures and coordinates of all structural models presented in this work are available from I.B. upon request.

### Molecular dynamics simulations of the S1–S2 linker

Molecular dynamic (MD) simulations of the S1–S2 linker (amino acids S748–D763) were done in the context of the S1–S4 sensor domain (i.e., amino acids S725–L850) which was surrounded with TIP3P water molecules (Jorgensen et al., 1983). This simplified simulated system was chosen to shorten the time for obtaining representative conformers of the S1–S2 linker. Amino acids S725–N747 and T764–L850 were fixed to avoid unfolding of the hydrophobic S1–S4 sensor domain due to the absence of phospholipids in our simulated system. The S1–S2 linker alone forms an extracellular loop. Therefore, its conformational dynamics was not biased by removing of phospholipids. We used the AMBER_ILDN force field (Lindorff-Larsen et al., 2010), which gives reliable results in molecular dynamics simulations of folding for both α-helical as well as β-sheet moieties. The software package GROMACS (Abraham et al., 2015) and Linux computer nodes with powerful NVIDIA GPUs enabled us to accumulate a 500 ns trajectory at 410 K.

### Molecular dynamics simulations of interactions between TRPA1 and Protoxin-I

To predict potential sites of interaction between TRPA1 and protoxin-I (Figures 8E,F), we took into account the homology of protoxin-I with the structurally similar vanillotoxin DkTx that binds the pore region of TRPV1 and traps it in its open state (Cao et al., 2013; Bae et al., 2016). We used again rigid docking using the SITUS software package (Wriggers et al., 1999) to place TRPA1 (i.e., amino acids A685-K989) and protoxin-I (2M9L) into a simulated electron map generated based on 3J5Q using VMD (see the “MDFF Tutorial” for details: http://www.ks.uiuc.edu/Training/Tutorials/). Resulting complex was embedded into the POPC phospholipidic bilayer and surrounded by TIP3P water molecules (Jorgensen et al., 1983) leading to a periodic box of size ~120 × ~120 × ~90 Å. 5-ns MD run produced by means of NAMD (Phillips et al., 2005) was used for relaxation. Finally, conformation II of the S1–S4 sensor module (gained within the MD simulation described above) was structurally aligned to produce Figures 8E,F.

### Channel constructs, cell culture and transfection

Human embryonic kidney 293T (HEK293T) cells were cultured in Opti-MEM I medium (Invitrogen) supplemented with 5% FBS as described previously (Benedikt et al., 2007). The day before transfection, cells were plated in 24-well plates (2 × 10^5^ cells per well) in 0.5 ml of medium and became confluent on the day of transfection. The cells were transiently co-transfected with 300 ng of cDNA plasmid encoding wild-type or mutant TRPA1 and with 200 ng of GFP plasmid (TaKaRa, Japan) using the magnet-assisted transfection technique (IBA GmbH, Goettingen, Germany) and then plated on poly-L-lysine-coated glass coverslips. The plasmid encoding human TRPA1 was in the pCMV6-XL4 vector (OriGene Technologies, Rockville, MD). The chicken TRPA1 in the pcDNA3.1 vector was kindly provided by Dr. Makoto Tominaga (National Institute of Natural Sciences, Okazaki, Aichi, Japan). At least three independent transfections were used for each experimental group. The wild-type channel was regularly tested in the same batch as the mutants. The cells were used 24–48 h after transfection. For biochemical studies, the plasmid encoding GFP-tagged human TRPA1 in the pCMV6-AC-GFP vector was used (OriGene Technologies, Rockville, MD). The mutants were generated by PCR using a QuikChange II XL Site-Directed Mutagenesis Kit (Agilent Technologies) and confirmed by DNA sequencing (GATC Biotech, Germany).

### Patch clamp recording

Whole-cell membrane currents were recorded by employing an Axopatch 200B amplifier and pCLAMP 10 software (Molecular Devices, Sunnyvalle, CA). Patch electrodes were pulled from a glass tube with a 1.5-mm outer diameter. The tip of the pipette was heat-polished, and its resistance was 3–5 MΩ. Series resistance was compensated by at least 70% in all recordings. The experiments were performed at room temperature (23–25°C). Only one recording was performed on any one coverslip of cells to ensure that recordings were made from cells not previously exposed to chemical stimuli. The extracellular control solution contained: 160 mM NaCl, 2.5 mM KCl, 1 mM CaCl_2_, 2 mM MgCl_2_, 10 mM HEPES, 10 mM glucose, adjusted to pH 7.3 with NaOH, 320 mOsm. The extracellular Ca^2+^-free solution (0 mM Ca^2+^) contained 150 mM NaCl, 10 mM HEPES, 2 mM HEDTA, adjusted to pH 7.3 with NaOH, 290 mOsm. The whole-cell pipette solution contained: 145 mM CsCl, 5 mM EGTA, 3 mM CaCl_2_, 10 mM HEPES, 2 mM MgATP, pH 7.3, adjusted with CsOH, 290 mOsm. Allyl isothiocyanate (AITC) solution was prepared prior to use from a 0.1 M stock solution in Me_2_SO. All of the chemicals were purchased from Sigma-Aldrich (St. Louis, MO). A system for rapid superfusion of the cultured cells was used for drug application (Dittert et al., 2006).

### Whole-cell lysate preparation and PNGase F treatment

HEK293T cells were transfected with 1.5 μg cDNA by Lipofectamine 2000 (Life Technologies) on a 6-well plate. After 48 h, cells were washed with ice-cold phosphate-buffered saline and collected. After brief centrifugation at 15,000 × g for 5 min at 4°C, cells were briefly sonicated in the presence of 10x denaturating buffer (provided by New England Biolabs together with PNGase F) supplemented with a protease inhibitor mixture (Roche Diagnostics). Cell lysate was briefly centrifuged and denaturated at 95°C for 10 min. The protein concentration was measured using BCA protein assay reagent. Peptide: The N-Glycosidase F (PNGase F) digestion of proteins was performed according to the manufacturer's instructions (New England Biolabs). 10x G7 reaction buffer, 10% NP-40 (v/v), and 1000 units of enzyme were added to 20 μg of proteins. The samples were incubated for 3 h at 37°C. After that, 4x sample buffer was added and samples were boiled at 95°C for 10 min. In all cases, a control sample was run in parallel (water was added instead of PNGase F). Samples digested with the enzyme and their controls were loaded side by side on a 7% SDS gel.

### Tunicamycin treatment

To study potential effects of N-glycosylation on TRPA1 channel functioning, transiently transfected HEK293T cells were grown in the presence of tunicamycin (5 μg/ml; Sigma Aldrich), an effective inhibitor of N-linked glycosylation, in mammalian cells for 24 h and then measured by the patch clamp technique. Stock of tunicamycin was prepared in Me_2_SO at 5 mg/ml. The vehicle was added to the control (i.e., untreated) cells. For each run of experiments an untreated control was done.

### SDS/PAGE and immunoblotting

Cell lysates of PNGase F-treated and untreated samples (control) of wild type or N747T/N753T mutant in 4x sample buffer were boiled for 5 min at 95°C. Similar amounts of proteins were applied and separated on 7% SDS/PAGE at 100 V ~3 h. The proteins were then transfered to a polyvinylidene difluoride (PVDF) membrane using a wet blotter at 60 V 3 h. The PVDF membrane was blocked with 5% non-fat milk in TBS-T buffer for 1 h and incubated on a shaker for 1 h with the primary antibody (mouse monoclonal GFP, 1:2000, OriGene). Three 10 min steps in TBS-T were used to wash out nonspecific bindings of the primary antibody. Horseradish-peroxidase-linked anti-mouse secondary IgG (Thermo Scientific; 1:10,000 dilution in 1% non-fat milk) was then added for 1 h at room temperature. After specific binding of secondary IgG, the membrane was washed 5-times for 5 min. The specific proteins were detected after incubating the membrane in an enhanced chemiluminescent HRP substrate kit (Thermo Scientific) on X-ray blue film (FOMA).

### Fluorometric Ca^2+^-imaging

Calcium imaging was done using transfected HEK293T cells on coverslips with the same amount (500 ng) of cDNA of wild-type human TRPA1, I751A/I752A, I751G/I752G mutant and GFP (200 ng) for 24–48 h after transfection. Before the experiment, cells were loaded with 4 μM Fura2-AM (Invitrogen) and 0.02% Pluronic acid F-127 (Invitrogen) dissolved in a control extracellular solution (160 mM NaCl, 2.5 mM KCl, 1 mM CaCl_2_, 2 mM MgCl_2_, 10 mM HEPES, 10 mM glucose, adjusted to pH 7.3 with NaOH, 320 mOsm) for 1 h followed by a 20-min wash in fresh bath solution. The Cell^∧^R imaging system was used to capture the fluorescence images obtained with alternating excitation at 340 and 380 nm and emission at >510 nm. Emission ratios (F_340_/F_380_) were calculated for each 0.5 s interval after subtraction of the background. A cellular response to an AITC was defined as an increase in the ratio by more than 0.3 during the 50-s application period. The AITC-induced responses of cells were normalized to their maximum responses evoked by 5 μM ionomycin. All experiments were performed at room temperature.

### Data analysis

Electrophysiological data were analyzed in pCLAMP10 (Molecular Devices). Curve fitting and statistical analyses were done in SigmaPlot 10 (Systat Software). Current- and conductance-voltage (*I*-*V* and *G*-*V*) relationships were obtained from steady-state whole-cell currents measured at the end of voltage steps measured from −80 to +200 mV in increments of +20 mV. Voltage-dependent gating parameters were estimated by fitting the conductance *G* = *I*/(*V*-*V*_rev_) as a function of test potential *V* to the Boltzmann equation: *G* = [(*G*_max_*-G*_min_)/(1+exp (- *zF*(*V*-*V*_1/2_)/*RT*))] + *G*_min_, where *z* is the apparent number of gating charges, *V*_1/2_ is the half-activation voltage, *G*_min_ and *G*_max_ are the minimum and maximum whole cell conductance, *V*_rev_ is the reversal potential, and *F, R*, and *T* have their usual thermodynamic meanings. Statistical significance was determined using Student's *t*-test or one-way analysis of variance, as appropriate. All data are presented as the means ± standard error of mean (SEM). Blots were analyzed with ImageJ software (NIH). The data measured by the Ca^2+^-imaging technique were collected and analyzed with the software Cell^∧^R (Olympus).

## Author note

During the preparation of this manuscript, a study was published showing that the S1–S2 extracellular loop of the TRPV6 channel contributes to interfaces between the S1–S4 sensor and pore domain (Saotome et al., 2016). Like in TRPA1, the S1–S2 loop in TRPV6 contains two tandems of hydrophobic residues contributing to domain interfaces.

## Author contributions

LM, IB, and VZ performed experiments; LM, IB, and VV analyzed data; VV, LM, and IB designed experiments and wrote the article. LM, IB, VZ, LZ, and VV revised the manuscript and approved the final version.

### Conflict of interest statement

The authors declare that the research was conducted in the absence of any commercial or financial relationships that could be construed as a potential conflict of interest. The reviewer SG and handling Editor declared their shared affiliation, and the handling Editor states that the process nevertheless met the standards of a fair and objective review.

## Supplementary material

Supplementary Figure 1 **Replacing isoleucines 751 and 752 with alanines or glycines markedly decreased AITC responses at hyperpolarized voltage. (A)** Summary bar graph of average amplitudes of AITC-induced responses (100 μM in the presence of Ca^2+^) for wild type (white bar, *n* = 13), I751A/I752A (green bar, *n* = 15) and I751G/I752G (red bar, *n* = 8) double mutants at positive (+70 mV) and negative potentials (−70 mV). **(B)** I751A/I752A and I751G/I752G mutant exhibited less basal activity and lower AITC-evoked Ca^2+^ influx. Time course of average changes in Fura-2 ratio (F_340_/F_380_) corresponding to intracellular Ca^2+^ level [Ca^2+^]_i_ induced by 50-s application of 100 μM AITC and then by 5 μM ionomycin in HEK293T cells transfected with wild type (WT, white circles, *n* = 30), I751A/I752A (green triangles, *n* = 29) and I751G/I752G (red squares, *n* = 47). The responses are normalized to ionomycin responses after a 90-s application. Double mutants exhibited lower basal activities (~0.2) than the wild type (~0.3). Data represent mean ± SEM. **(C)** Statistical analysis of maximal AITC-induced responses normalized to ionomycin responses for wild type (white bar, *n* = 30), I751A/I752A (green bar, *n* = 29) and I751G/I752G (red bar, *n* = 47). Data represent mean ± SEM. The asterisks indicate significant difference from wild-type TRPA1; ^*^*p* < 0.05; ^**^*p* < 0.001.

Supplementary Figure 2 **Small residues at positions of isoleucines 751 and 752 prevent TRPA1 activation by AITC especially in the absence of Ca^2+^ at negative potentials. (A–E)** All the double mutants were examined using a saturated concentration of AITC (100 μM) in the absence (30 s, white bar) or presence of Ca^2+^ (1 mM) in combination with a voltage ramp protocol (as in Figure 4C). For I751F/I752F (*n* = 10), I751P/I752P (*n* = 9) and I751W/I752W (*n* = 9), no significant changes were observed in the peak of AITC-induced responses, either in the absence or presence of Ca^2+^ at both membrane potentials (−70 mV, +70 mV). For comparison, the wild type currents are depicted in the background (white circles, *n* = 63). Data represent mean ± SEM. **(F)** Specific combination of volume, hydrophobicity and flexibility of amino acids at the positions of isoleucines 751 and 752 is critical for voltage-dependent gating of TRPA1. The relationship between voltage sensitivity (x axis; current responses evoked by +200 mV in the control extracellular solution) and the maximum AITC-induced responses for wild-type TRPA1 (II, white circle, *n* = 63) and double-isoleucine mutants, measured at +70 mV (y axis; 100 μM AITC in control extracellular solution). Hydrophobicities for the residues are depicted: phenylalanine (I751F/I752F, brown diamond, *n* = 17), the big amino acid with the highest hydrophobicity (volume 135 Å/hydrophobicity score 3.7), smaller and less hydrophobic residues alanine (67 Å/1.6) and glycine (48 Å/1.0) (green triangle, *n* = 22, and red square, *n* = 31), tryptophan (I751W/I752W, blue triangle, *n* = 21) and proline (I751P/I752P, yellow square, *n* = 10). All data here represent mean ± SEM.

Supplementary Figure 3 **N747T/N753T mutations caused decreased expression of TRPA1 channels. (A)** Representative current traces in response to indicated voltage step protocol of wild type and N747T/N753T double mutant. **(B)** Average conductances obtained from voltage-step protocols as in **(A)** for wild type (white circles, *n* = 121) and mutant (blue squares, *n* = 17). **(C)** Average AITC currents in the absence (white horizontal bar) and subsequently in the presence of 1 mM Ca^2+^ in extracellular solution (black horizontal bar), measured at −70 and +70 mV. AITC was applied for the time indicated by the horizontal bar. Wild-type current responses are indicated with white circles, wild type treated with tunicamycin (5 μg/ml, 24 h) with orange triangles and N747T/N753T double mutant with blue squares. **(D)** Changes in the rectification index (*R* = −I_−70mV_/I_+70mV_) plotted as a function of time. **(E)** Immunoblot of total protein profile of wild type and N747T/N753T mutant in transiently transfected HEK293T cells treated with PNGase F (indicated with +) and their non-digested control samples (indicated with −). The same amount of proteins from the cell lysate was used in each sample. **(F)** Summary bar graph shows average band intensity for wild type (*n* = 16) and N747T/N753T mutant (*n* = 16). Asterisks indicate a significant difference from wild-type TRPA1, ^*^*p* < 0.05. Data represent mean ± SEM (n in brackets).

Supplementary Figure 4 **Chicken TRPA1 exhibits slow activation kinetics**. Representative current response recorded from HEK293T cell expressing chicken TRPA1 (cTRPA1) activated with 100 μM AITC in changing Ca^2+^ concentration at potential −70 mV. The applications of AITC with (black horizontal bar) and without Ca^2+^ ions (1 mM, white horizontal bar) are indicated above the current trace.

Video 1 **Modeling the open state of TRPA1 channel**. Model with each of the four identical subunits color-coded, showing views from side and top during channel opening. The lower gate-forming residues (I957 and V961) and position of proline P742 (yellow, S1–S2 linker) relative to three hydrophobic residues V737 (S1), W832 (S4) and I890 (S5) forming a “hydrophobic switch” (salmon) are explicitely shown in details. See also Figure 2.
