# Supplementary material for: The First Extracellular Linker Is Important for Several Aspects of the Gating Mechanism of Human TRPA1 Channel
Source: Front Mol Neurosci. 2017 Jan 31;10:16. doi: 10.3389/fnmol.2017.00016 (PMC5281607; doi:10.3389/fnmol.2017.00016)
Supplement: Supplementary Figure 1 — Replacing isoleucines 751 and 752 with alanines or glycines markedly decreased AITC responses at hyperpolarized voltage. (A) Summary bar graph of average amplitudes of AITC-induced responses (100 μM in the presence of Ca2+) for wild type (white bar, n = 13), I751A/I752A (green bar, n = 15) and I751G/I752G (red bar, n = 8) double mutants at positive (+70 mV) and negative potentials (−70 mV). (B) I751A/I752A and I751G/I752G mutant exhibited less basal activity and lower AITC-evoked Ca2+ influx. Time course of average changes in Fura-2 ratio (F340/F380) corresponding to intracellular Ca2+ level [Ca2+]i induced by 50-s application of 100 μM AITC and then by 5 μM ionomycin in HEK293T cells transfected with wild type (WT, white circles, n = 30), I751A/I752A (green triangles, n = 29) and I751G/I752G (red squares, n = 47). The responses are normalized to ionomycin responses after a 90-s application. Double mutants exhibited lower basal activities (~0.2) than the wild type (~0.3). Data represent mean ± SEM. (C) Statistical analysis of maximal AITC-induced responses normalized to ionomycin responses for wild type (white bar, n = 30), I751A/I752A (green bar, n = 29) and I751G/I752G (red bar, n = 47). Data represent mean ± SEM. The asterisks indicate significant difference from wild-type TRPA1; *p < 0.05; **p < 0.001. [file DataSheet1.DOC]

Supplementary Material

The first extracellular linker is important for several aspects of the gating mechanism of human TRPA1 channel

**Lenka Marsakova, Ivan Barvik, Vlastimil Zima, Lucie Zimova, Viktorie Vlachova ^1*^**

*** Correspondence:** Viktorie Vlachova: Viktorie.vlachova@fgu.cas.cz

**
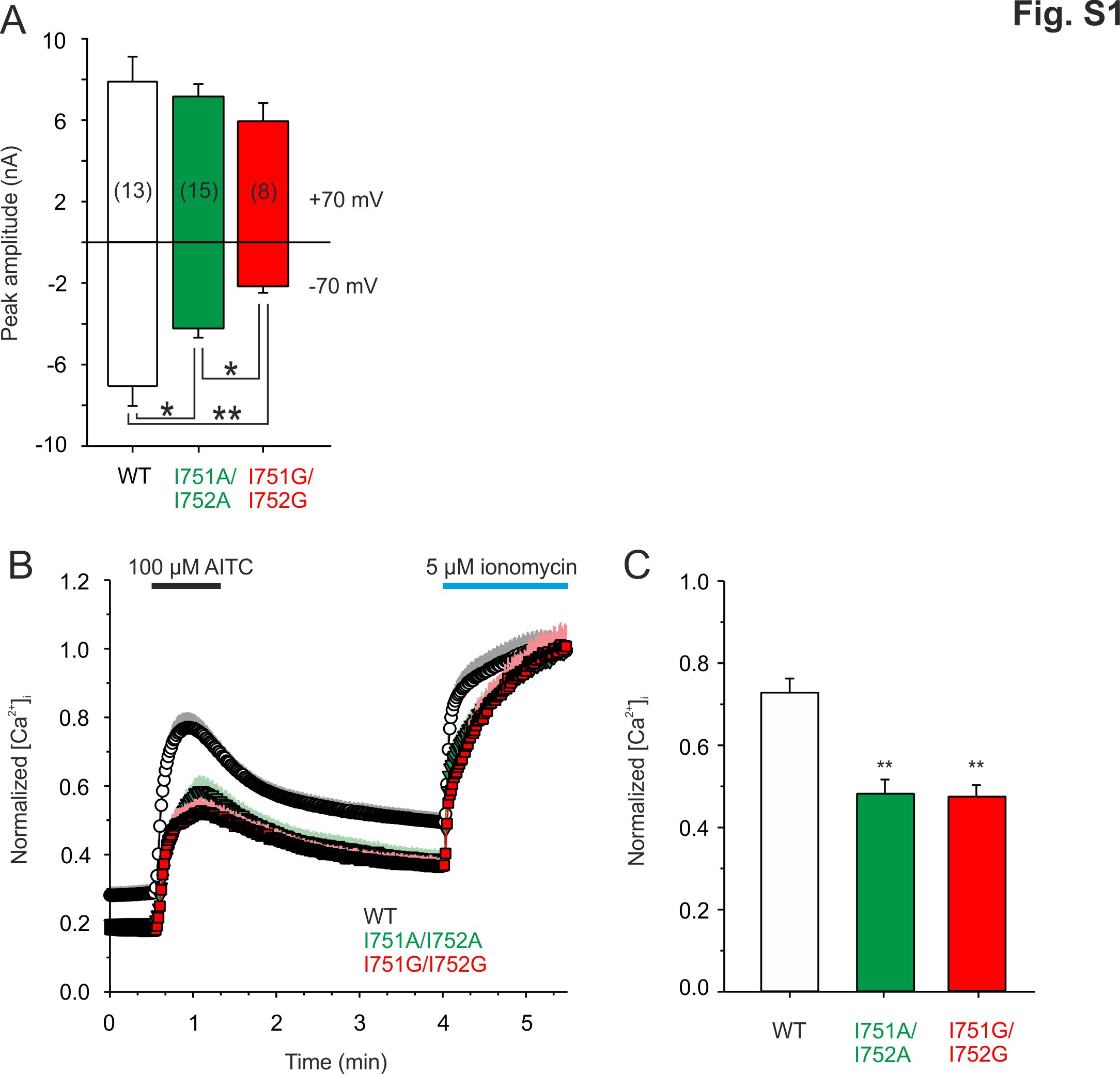
**

**Supplementary Figure 1.** Replacing isoleucines 751 and 752 with alanines or glycines markedly decreased AITC responses at hyperpolarized voltage. (A) Summary bar graph of average amplitudes of AITC-induced responses (100 μM in the presence of Ca^2+^) for wild type (white bar, n = 13), I751A/I752A (green bar, n = 15) and I751G/I752G (red bar, n = 8) double mutants at positive (+70 mV) and negative potentials (-70 mV). (B) I751A/I752A and I751G/I752G mutant exhibited less basal activity and lower AITC-evoked Ca^2+^ influx. Time course of average changes in Fura-2 ratio (F_340_/F_380_) corresponding to intracellular Ca^2+^ level [Ca^2+^]_i_ induced by 50-s application of 100 μM AITC and then by 5 μM ionomycin in HEK293T cells transfected with wild type (WT, white circles, n = 30), I751A/I752A (green triangles, n = 29) and I751G/I752G (red squares, n = 47). The responses are normalized to ionomycin responses after a 90-s application. Note, double mutants exhibited lower basal activities (~0.2) than the wild type (~0.3). Data represent mean ± SEM. (C) Statistical analysis of maximal AITC-induced responses normalized to ionomycin responses for wild type (white bar, n = 30), I751A/I752A (green bar, n = 29) and I751G/I752G (red bar, n = 47). Data represent mean ± SEM. The asterisks indicate significant difference from wild-type TRPA1; *p<0.05; **p<0.001.


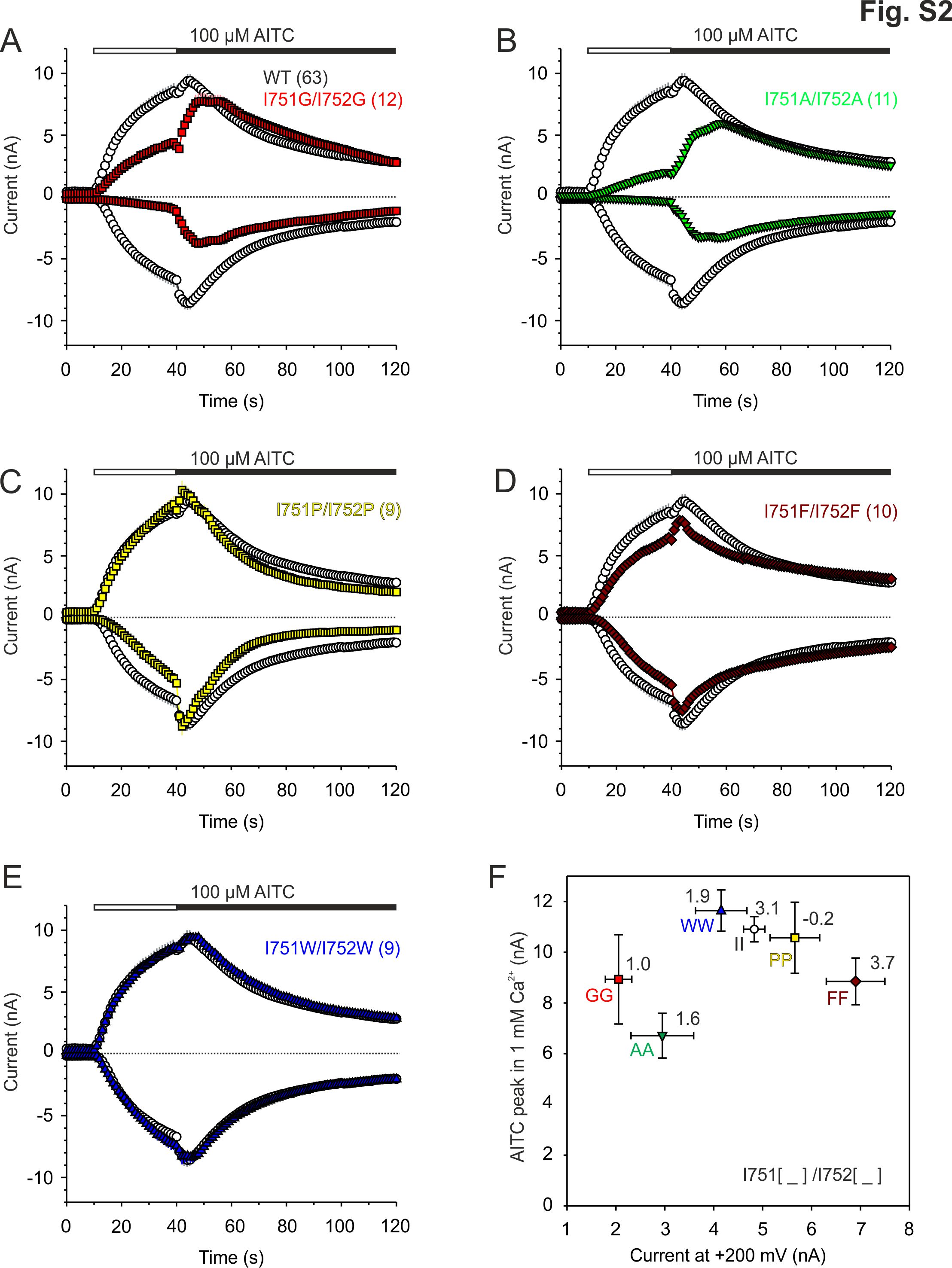


**Supplementary Figure 2.** Small residues at positions of isoleucines 751 and 752 prevent TRPA1 activation by AITC especially in the absence of Ca^2+^ at negative potentials. (A-E) All the double mutants were examined using a saturated concentration of AITC (100 μM) in the absence (30 s, white bar) or presence of Ca^2+^ (1 mM) in combination with a voltage ramp protocol (as in Fig. 4C). For I751F/I752F (n = 10), I751P/I752P (n = 9) and I751W/I752W (n = 9), no significant changes were observed in the peak of AITC-induced responses, either in the absence or presence of Ca^2+^ at both membrane potentials (-70 mV, +70 mV). For comparison, the wild type currents are depicted in the background (white circles, n = 63). Data represent mean ± SEM. (F) Specific combination of volume, hydrophobicity and flexibility of amino acids at the positions of isoleucines 751 and 752 is critical for voltage-dependent gating of TRPA1. The relationship between voltage sensitivity (x axis; current responses evoked by +200 mV in the control extracellular solution) and the maximum AITC-induced responses for wild-type TRPA1 (II, white circle, n = 63) and double-isoleucine mutants, measured at +70 mV (y axis; 100 μM AITC in control extracellular solution). Hydrophobicities for the residues are depicted : phenylalanine (I751F/I752F, brown diamond, n = 17), the big amino acid with the highest hydrophobicity (volume 135 Å/hydrophobicity score 3.7), smaller and less hydrophobic residues alanine (67 Å /1.6) and glycine (48 Å/1.0) (green triangle, n = 22, and red square, n = 31) , tryptophan (I751W/I752W, blue triangle, n = 21) and proline (I751P/I752P, yellow square, n = 10). All data here represent mean ± SEM.


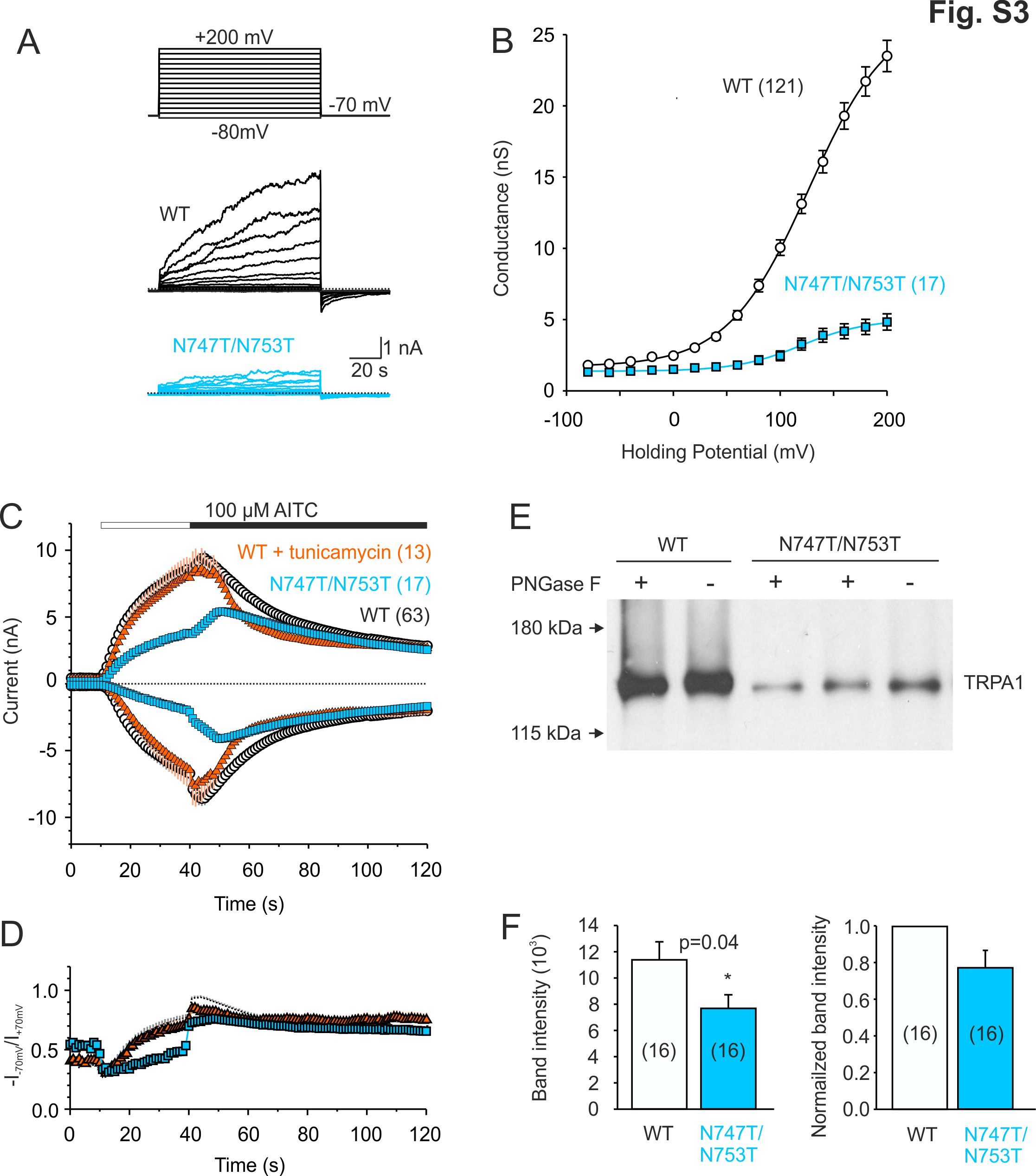


**Supplementary Figure 3.** N747T/N753T mutations caused decreased expression of TRPA1 channels. (A) Representative current traces in response to indicated voltage step protocol of wild type and N747T/N753T double mutant. (B) Average conductances obtained from voltage-step protocols as in A for wild type (white circles, n = 121) and mutant (blue squares, n = 17). (C) Average AITC currents in the absence (white horizontal bar) and subsequently in the presence of 1 mM Ca^2+^ in extracellular solution (black horizontal bar), measured at -70 and +70 mV. AITC was applied for the time indicated by the horizontal bar. Wild-type current responses are indicated with white circles, wild type treated with tunicamycin (5 μg/ml, 24 h) with orange triangles and N747T/N753T double mutant with blue squares. (D) Changes in the rectification index (R = -I_-70mV_/I_+70 mV_) plotted as a function of time . (E) Immunoblot of total protein profile of wild type and N747T/N753T mutant in transiently transfected HEK293T cells treated with PNGase F (indicated with +) and their non-digested control samples (indicated with -). The same amount of proteins from the cell lysate was used in each sample. (F) Summary bar graph shows average band intensity for wild type (n = 16) and N747T/N753T mutant (n = 16). Asterisks indicate a significant difference from wild-type TRPA1, *p<0.05. Data represent mean ± SEM (n in brackets).


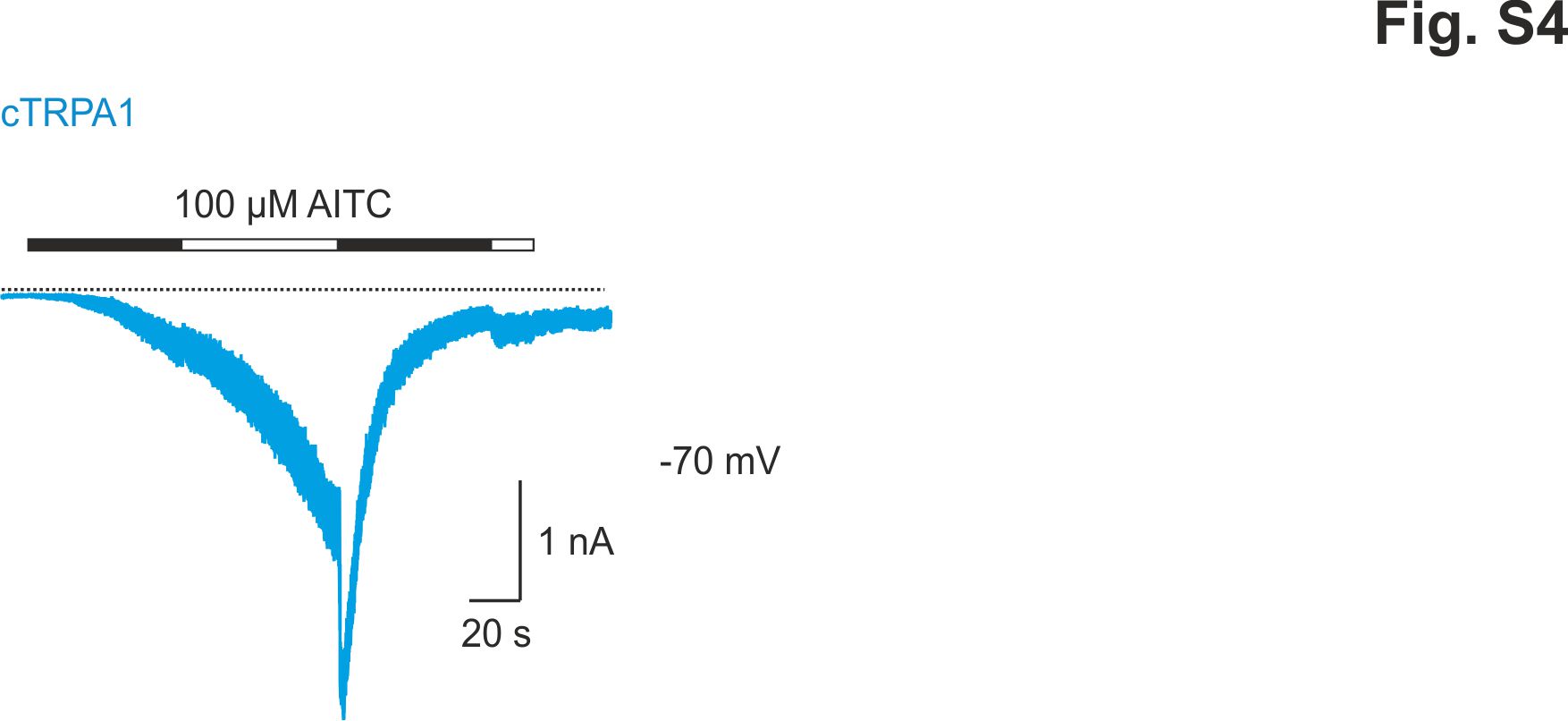


**Supplementary Figure 4.** Chicken TRPA1 exhibits slow activation kinetics. Representative current response recorded from HEK293T cell expressing chicken TRPA1 (cTRPA1) activated with 100 μM AITC in changing Ca^2+^ concentration at potential -70 mV. The applications of AITC with (black horizontal bar) and without Ca^2+^ ions (1 mM, white horizontal bar) are indicated above the current trace.
